# Overview of the immunomodulatory role of bacterial probiotic-derived peptidoglycan: from molecular insights to therapeutic application

**DOI:** 10.3389/fmicb.2026.1761985

**Published:** 2026-02-26

**Authors:** Omer Qutaiba B. Allela, Abdulkareem Shareef, Hayder Naji Sameer, Ahmed Yaseen, Zainab H. Athab, Mohaned Adil

**Affiliations:** 1 College of Pharmacy, Alnoor University, Mosul, Iraq; 2 Ahl Al Bayt University, Kerbala, Iraq; 3 College of Pharmacy, National University of Science and Technology, Muscat, Iraq; 4 Gilgamesh Ahliya University, Baghdad, Iraq; 5 Department of Pharmacy, Al-Zahrawi University College, Karbala, Iraq; 6 Pharmacy College, Al-Farahidi University, Baghdad, Iraq

**Keywords:** cancer, immune modulation, infection, inflammation, inflammatory bowel disease (IBD), nucleotide-binding oligomerization domain (NOD) receptor, probiotic-derived peptidoglycan, toll-like receptor (TLR)

## Abstract

Probiotics are well recognized for their ability to modulate host immune responses; however, growing evidence indicates that many of their beneficial effects are mediated by structural components rather than by viable microorganisms. Among these components, probiotic-derived peptidoglycan has emerged as a key immunologically active molecule with a critical role in regulating both innate and adaptive immunity. Although substantial experimental data exist regarding its underlying mechanisms, the context-dependent immunomodulatory and anti-inflammatory functions of peptidoglycan have not been comprehensively integrated. In this review, we provide an up-to-date and comprehensive overview of the molecular and cellular mechanisms that govern the immunoregulatory properties of probiotic-derived peptidoglycan. We first discuss the structural diversity and processing of peptidoglycan and their implications for host recognition via pattern-recognition receptors, particularly Toll-like receptor 2 (TLR2) and nucleotide-binding oligomerization domain proteins 1 and 2 (NOD1/2). We then critically evaluate current evidence supporting the therapeutic potential of probiotic-derived peptidoglycan in infectious diseases, inflammatory bowel disease (IBD), autoimmune disorders, allergic inflammation, and cancer. Collectively, these findings suggest that peptidoglycan holds considerable promise for the development of next-generation microbiota-based immunotherapeutic strategies.

## 1 Introduction

Peptidoglycan, also known as murein, mucopeptide, or mucocomplex, is the bacterial cell wall building block that provides cell shape and protection from osmotic pressure (Garde et al., 2021). Peptidoglycan is a thin layer of cells in Gram-negative bacteria and a thicker cell layer in Gram-positive bacteria (Vollmer et al., 2008; Garde et al., 2021). Current research has emphasized the functional significance of peptidoglycan fragments as signaling molecules that modulate inflammatory processes upon host immune recognition (Irazoki et al., 2019; Bastos et al., 2021). Peptidoglycan functions as a key microbe-associated molecular pattern (MAMP) that elicits immunostimulatory responses through interaction with pattern-recognition receptors (PRRs) (Lee et al., 2023). The receptors identify peptidoglycan derivatives to initiate subsequent signal transduction cascades that eventually can result in immune cell recruitment and inflammatory responses for the elimination of microorganisms (Lee et al., 2023). Peptidoglycan derived from pathogenic bacteria is classically associated with pro-inflammatory immune activation, promoting cytokine production and innate immune defense (Lin et al., 2010). However, accumulating evidence indicates that peptidoglycan originating from commensal and probiotic bacteria exerts distinct and often regulatory immunological effects, highlighting the context-dependent nature of peptidoglycan-mediated signaling (Sun et al., 2005).

In addition, a recent study found that *Lactobacillus acidophilus* peptidoglycan-disaccharide-bound fragments can stimulate macrophages into proinflammatory cytokine production (Bersch et al., 2021). Treatment of lipopolysaccharide (LPS)-stimulated RAW 264.7 macrophages with *L. acidophilus*–derived peptidoglycan resulted in decreased expression of cyclooxygenase-2 (COX-2) and inducible nitric oxide synthase (iNOS), critical mediators of inflammation (Wu et al., 2013). *Lactobacillus* peptidoglycan has been shown to suppress interleukin-12 (IL-12) production, thereby limiting downstream induction of interferon-*γ* (IFN-γ) and tumor necrosis factor-*α* (TNF-α), cytokines central to T-cell–mediated inflammatory responses (Shida et al., 2009).

Sensing of muramyl dipeptide (MDP), a peptidoglycan-derived compound, by nucleotide-binding oligomerization domain 2 (NOD2) promotes receptor-interacting protein kinase 2 (RIP2) recruitment and activation, which in turn initiates nuclear factor-κB (NF-κB)- and mitogen-activated protein kinase (MAPK)-dependent signaling pathways (Magalhaes et al., 2011). Importantly, NOD2 signaling contributes to host defense while simultaneously restraining excessive Toll-like receptor (TLR)–mediated inflammation, thereby preventing uncontrolled cytokine production. This dual role provides a mechanistic basis for the anti-inflammatory effects observed following stimulation with probiotic-derived peptidoglycan. Consistently, MDP has been identified as an anti-inflammatory, insulin-sensitizing postbiotic acting through NOD2 activation. In parallel, *Lactobacillus*-derived peptidoglycan promotes the maturation of CD103^+^ dendritic cells (DCs) and enhances CD4^+^ Forkhead box P3 (Foxp3)^+^ regulatory T cells (Tregs) differentiation, further confirming its immunoregulatory function (Fernandez et al., 2011). This review summarizes the molecular mechanism of immunomodulation by peptidoglycan derived from probiotics and outlines prospective therapeutic avenues of intervention in health and disease.

## 2 Probiotics and probiotics-derived effector molecules

Probiotics are defined by the Food and Agriculture Organization and the World Health Organization as live microorganisms that, when administered in adequate amounts, confer a health benefit on the host (Hill et al., 2014). Their potential health benefits are thought to arise from multiple mechanisms, including enhancement of intestinal barrier integrity, inhibition or competitive exclusion of pathogens, promotion of beneficial symbionts, and modulation of both local and systemic immune responses (Lebeer et al., 2008). During the last decades, a series of research investigations have tried to establish the effectiveness of probiotics in numerous illnesses, including the reduction of inflammatory bowel disease (IBD) and cardiovascular disease, depression symptoms, protection from or treatment of atopic dermatitis, and infections (Muzaffar et al., 2021). The most common probiotics consumed in human nutrition include the genera *Lactobacillus*, *Bacillus*, *Bifidobacterium*, and *Saccharomyces* (Muzaffar et al., 2021). Among these, lactic acid bacteria (LAB) are the most commonly used probiotic microorganisms in the form of viable cells. Homofermentative *Lactobacillus* species are grouped into three large categories—*L. acidophilus*, *Lactobacillus salivarius*, and *Lactobacillus rhamnosus* are of particular prominence (Muzaffar et al., 2021). Probiotic LAB have also demonstrated great potential as a replacement for antibiotics, playing preventive and therapeutic roles in several health disorders (Muzaffar et al., 2021). In this context, it is important to distinguish probiotics from postbiotics. Probiotics refer exclusively to viable microorganisms, whereas postbiotics are defined as non-viable microbial cells, cell fragments, or metabolites that confer health benefits to the host (Vinderola et al., 2022). Probiotics-derived effector molecules, including peptidoglycan, fall within the postbiotic category and can reproduce many of the immunomodulatory effects attributed to live probiotic bacteria (Wang et al., 2024).

More recent evidence has shown that live-growing bacteria are not necessary to induce beneficial effects (Ji et al., 2023). Experimental evidence has demonstrated that inactivated bacteria, vesicles derived from bacteria, or individual effector molecules can elicit similar beneficial effects (Ji et al., 2023). These encompass surface-attached components such as peptidoglycan and polysaccharides, secreted antibacterial peptides, and bioactive molecules from bacterial lysis, including proteins, exopolysaccharides, and DNA (Ji et al., 2023). Surface-associated effector molecules, such as cellular constituents or metabolic products secreted into the environment, could impart several health-promoting attributes (Bhawal et al., 2023). Bacterial cell wall structures are effector ligands that enable host-bacteria interaction and host receptor initiation of signaling pathways that are responsible for the health effects of probiotics (Bhawal et al., 2023). These effector molecules have been shown to regulate immune homeostasis between T helper (Th) 17 and Tregs activity by new mechanisms, with some of the mechanisms of action of the molecules showing proinflammatory activities and others associated with anti-inflammatory pathways (Lee et al., 2023). Improved molecular and cellular insight into the immunological effects of individual probiotic components will facilitate more reliable prediction of immune outcomes in future probiotic clinical studies, considering study conditions and combined effector mechanisms.

## 3 Immunomodulatory function of probiotics-derived peptidoglycan

**Figure 1:**
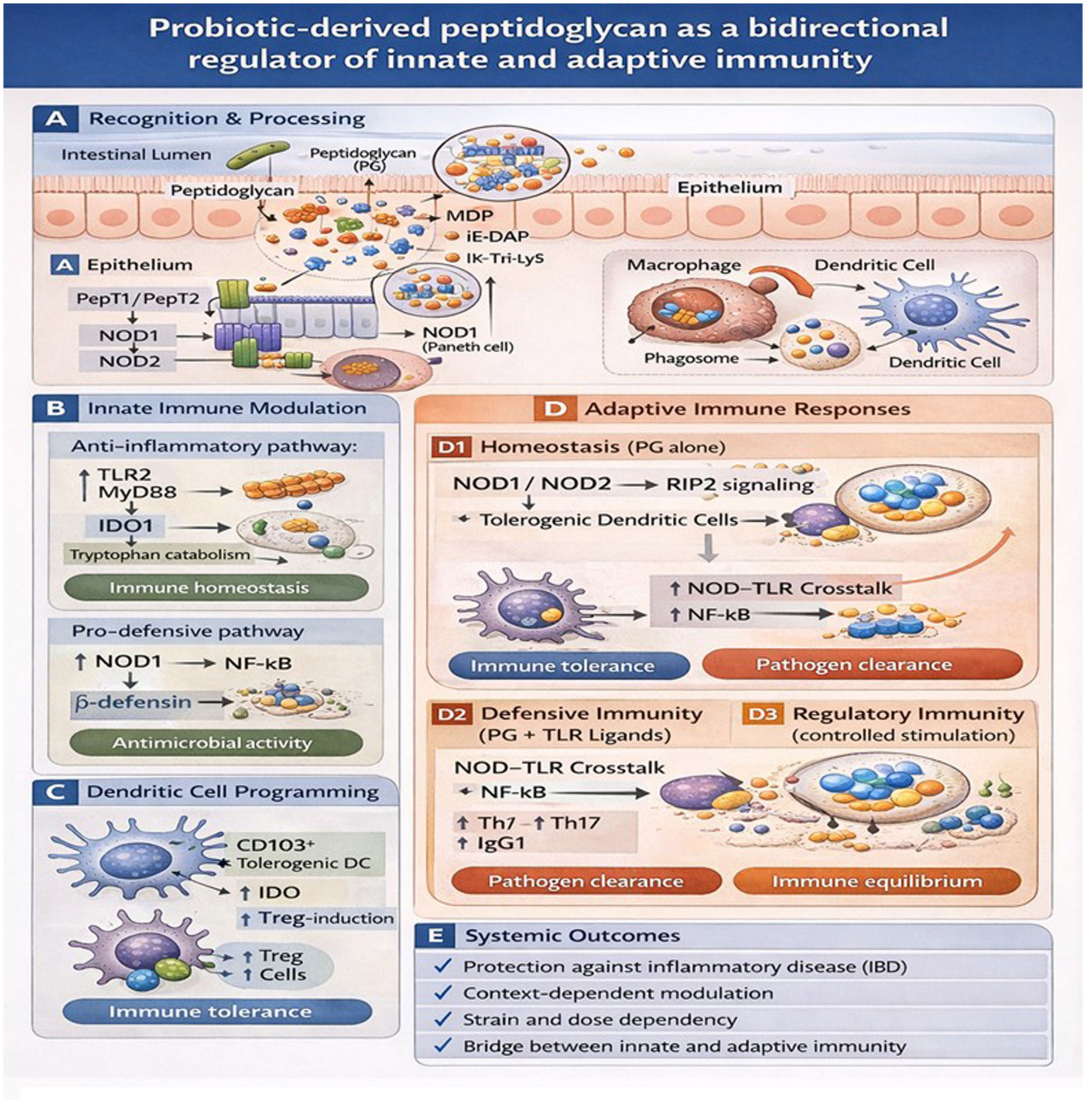
Probiotic-derived peptidoglycan (PG) as a bidirectional regulator linking innate and adaptive immunity. **(A)** PG fragments released in the intestinal lumen are transported across the epithelium via PepT1/PepT2 and sensed intracellularly by NOD1 and NOD2 in epithelial and antigen-presenting cells. Phagocytic uptake by macrophages and dendritic cells further processes PG into immunologically active muropeptides. **(B)** In innate immune modulation, PG activates two context-dependent pathways: a TLR2-MyD88-IDO1 axis promoting tryptophan catabolism and immune homeostasis, and a NOD1-NF-κB pathway inducing antimicrobial peptide production (e.g., *β*-defensins). **(C)** PG-exposed dendritic cells acquire a CD103^+^ tolerogenic phenotype, up-regulate indoleamine-2,3-dioxygenase (IDO), and promote regulatory T-cell induction. **(D)** Adaptive immune responses depend on the inflammatory context. In the absence of strong TLR stimulation (PG alone), NOD1/NOD2-RIP2 signaling promotes tolerogenic dendritic cells, expansion of CD4^+^CD25^+^FoxP3^+^ Treg cells, IL-10 production, and mucosal tolerance. When PG signaling coincides with TLR ligands, NOD-TLR crosstalk activates NF-κB, leading to Th1/Th17 differentiation, IgG1 production, and pathogen clearance. Under controlled stimulation, regulatory immunity maintains a balanced Treg/effector response and immune equilibrium. **(E)** Collectively, these mechanisms explain how probiotic-derived PG bridges innate and adaptive immunity, providing protection against inflammatory disease, while its effects remain strain-, dose-, and context-dependent.

The results identify the function of probiotic-derived peptidoglycan in facilitating active host immune cell-probiotic communication (Figure 1). In this regard, the factors are involved in the regulation of immune signaling, which modulates innate immune responses under healthy conditions and adjusts immunity under disease conditions (Table 1).

**Table 1:** Immunomodulatory role of probiotic-derived peptidoglycan in health and disease.

Probiotic strain	Study setting	Immune function	Mechanism	Outcome	References
*Lactobacillus rhamnosus* MLGA	*In vitro*	Enhances innate immunity without inflammation	Peptidoglycan upregulates avian β-defensin 9 (a key antimicrobial peptide) without inducing IL-1β, IL-8, or IL-12p40	Increased antimicrobial activity against *Salmonella Enteritidis* while avoiding excessive inflammation	Huang et al. (2020)
*Bifidobacterium* spp. (Paraimmunobiotic strains)	*In vitro*	Modulates PRR signaling, enhances resistance to infections, and reduces inflammatory damage	Peptidoglycan recognition proteins (PGLYRPs) are upregulated in a strain-dependent manner, interacting with TLR2, TLR4, NOD1, and NOD2 pathways	Potential use in reducing the severity of inflammatory diseases and improving resistance to intestinal infections	Iida et al. (2019)
*Lactobacillus* spp.	*In vivo*	Enhancement of innate immune sensing; regulation of gut immune homeostasis; anti-inflammatory protection; tumor-suppressive immunity in inflammation-driven CRC	Secreted LPH (*Lactobacillus* Peptidoglycan Hydrolase) acts as a bi-functional peptidoglycan hydrolase (N-acetyl-β-D-muramidase + DL-endopeptidase), generating muramyl dipeptide (MDP) from bacterial peptidoglycan → MDP activates NOD2 signaling in host cells; effects are abolished with LPH active-site mutants or in Nod2-deficient mice	Protection against experimental colitis; reduced intestinal inflammation; maintenance of gut homeostasis; attenuation of inflammation-associated colorectal cancer in a female-specific manner	Gao et al. (2022)
*Bifidobacterium longum; Clostridium butyricum; Lactobacillus plantarum WCFS1*	*In vitro*	Activation of innate immune responses; adjuvant-like immunostimulation via macrophages and dendritic cells	Probiotic-derived extracellular vesicles (EVs) (100–150 nm) contain peptidoglycan that activates host immune sensing; EVs are internalized mainly via clathrin-mediated endocytosis and macropinocytosis; EV-associated peptidoglycan induces cytokine production	Induction of inflammatory cytokines (TNF-α, IL-6) from macrophages and dendritic cells (DCs); identification of probiotic-derived EVs as novel immune adjuvants for EV-based immunotherapy	Morishita et al. (2021)
*Lactobacillus acidophilus*	*In vitro*	Anti-inflammatory, immunomodulatory	Phosphorylated peptidoglycan reduces GM-CSF, TNF-α, and IL-1 secretion in a dose-dependent manner	Suppressed inflammation in LPS-stimulated macrophages, suggesting potential therapeutic applications	Wu et al. (2016)
*L. acidophilus* IMV B-7279, *Lactobacillus casei* IMV B-7280, *L. delbrueckii subsp. bulgaricus* IMV B-7281, *Bifidobacterium animalis* VKL, VKB	*In vivo*	Enhances macrophage activity, NO production, and cytokine release	Bacterial wall elasticity affects intracellular digestion and immune activation; strains with higher elasticity survive longer inside macrophages	Higher IL-12 and IFN-γ production, stronger stimulation of macrophages, potential for immune modulation in infections.	Mokrozub et al. (2013)
*Lactobacillus salivarius* Ls33	*In vivo*	Protects against colitis, induces regulatory T cells (Tregs) and tolerogenic DCs	Peptidoglycan-derived muropeptide (M-tri-Lys) activates NOD2 signaling, increasing IL-10 and regulatory CD103 + DCs	Protection against colitis was NOD2-dependent and correlated with IL-10 production, FoxP3 + Tregs, and IDO activation.	Fernandez et al. (2011)
*Bifidobacterium* spp.*; Lactobacillus* spp.	*In vitro*	TLR-dependent innate immune activation; amplification of proinflammatory signaling	EV-associated peptidoglycan recognized by TLR2; cytokine induction reduced by TLR2-blocking antibody (T2.5); downstream activation of JNK/MAPK and NF-κB signaling pathways	Robust induction of TNF-α and IL-6 via TLR2–MAPK–NF-κB axis; mechanistic validation of probiotic EVs as TLR2-mediated immune adjuvants	Morishita et al. (2022)
*L. casei ATCC 393*	*In vivo*	Anti-inflammatory immune modulation; intestinal barrier protection; microbiota-mediated immune regulation	Dietary peptidoglycan supplementation modulates gut microbiota composition (↑ *Firmicutes/Bacteroidetes* ratio; ↑ *Lachnospiraceae* and *Lactobacillus*); enhances microbial metabolic activity → increased SCFA production (propionate, butyrate); suppression of systemic inflammatory cytokines	Reduced disease activity index, weight loss, and colon shortening; improved epithelial barrier integrity; decreased serum IL-6, TNF-α, IL-1β; protection against DSS-induced colitis via microbiota–SCFA–immune axis	Li et al. (2025)
*L. plantarum* microspheres	*In vivo*	Enhances intestinal microbiota balance and systemic immune response	Upregulates peptidoglycan recognition protein (PGRP) expression in multiple organs, increases cytokine levels (IL-4, IL-12, IFN-γ, TNF-α)	Increased *Lactobacillus* and *Bifidobacterium* levels in cecum, improved systemic Th1/Th2 balance, potential use as adjuvant	Li X. et al. (2017)
*L. acidophilus* (Peptidoglycan-derived disaccharides)	*In vitro*	Differentially regulates innate immune signaling	Synthetic peptidoglycan disaccharides and monosaccharides activate NOD-like receptors and PRRs, inducing distinct inflammatory responses	Disaccharide peptidoglycan units differentially modulate immune signaling compared to classical MDP, highlighting complexity in Peptidoglycan-immune interactions	Bersch et al. (2021)
*Lactobacillus rhamnosus BBMN68*	*In vivo* and *in vitro*	Immunomodulatory/postbiotic/microbiota modulation	Peptidoglycan identified as key bioactive; enhanced Th1/Th2 balance, increased serum IgA, IgG, IgM, hemolysin; modulated cytokines (↑IL-2, TNF-α, IFN-γ, IL-4, IL-6; ↓IL-1β, IL-10); altered gut microbiota (↑Lachnospiraceae_NK4A136, Dubosiella; ↓Clostridium_sensu_stricto_1, Faecalibaculum, Romboutsia); suppressed TNF-α and IL-1β mRNA in RAW264.7 cells	Mitigated body weight loss, improved immune organ indices and hematopoietic function, enhanced delayed-type hypersensitivity responses, strengthened host immunity; safer and more effective than Levamisole	Zhou et al. (2025)
*L. casei Shirota (LcS)*	Murine IBD and colitis-associated cancer models and *in vitro*	Anti-inflammatory/tumor-suppressive	Inhibition of IL-6 production via NF-κB suppression, blocking IL-6/STAT3 signaling	Improved murine IBD (reduced ileitis), suppressed colitis-associated cancer; LC(ΔPSPG-I) mutant ineffective	Matsumoto et al. (2009)
*Enterococcus faecium*	*In vitro* and *in vivo*	Promotion of intestinal immunity and cancer immunotherapy efficacy; bacterial fitness and stress resistance	SagA, a conserved peptidoglycan hydrolase, generates bioactive muropeptides → NOD2 activation; ΔsagA reduces muropeptide release, NOD2 signaling, and immune checkpoint inhibitor efficacy; catalytically inactive SagA fails to restore function	Loss of SagA impairs bacterial growth, cell separation, antibiotic tolerance, NOD2 activation, and antitumor immunity; functional SagA restores immune stimulation	Klupt et al. (2024)
*Bifidobacterium* spp.	*In silico* and *in vitro*	Discovery of novel anti-inflammatory peptidoglycan fragments; advancement of peptidoglycan profiling methodology	Development of peptidoglycan _MS2, an in silico MS/MS prediction tool enabling automated identification of peptidoglycan; identification of abundant 1,6-anhydro-MurNAc–containing peptidoglycan in *Bifidobacterium* spp.; production mediated by lytic transglycosylases (LTs); anhydro-peptidoglycan exhibits direct anti-inflammatory activity *in vitro*	High-resolution mapping of gut peptidoglycan diversity; identification of previously unrecognized bioactive peptidoglycan; establishment of *Bifidobacterium*-derived anhydro-peptidoglycan as immunoregulatory molecular signals in microbiota–host crosstalk	Kwan et al. (2024)
*Lacticaseibacillus rhamnosus and L. acidophilus* (PGN-Lr, PGN-La)	*In vitro*	Anti-inflammatory; suppression of pathogen-induced uterine inflammation	Lactic acid bacteria (LAB) -derived PG shares GlcNAc–MurNAc backbone but contains distinct cross-bridge modifications (likely D-aspartate instead of glycine); peptidoglycan -L does not activate TLR2/1 alone; pretreatment antagonizes PAM3CSK4-induced TLR2/1 signaling, reducing TNF, IL1B, CXCL8, PTGES expression and PGE2 secretion; in silico modeling suggests interference with TLR2/1 heterodimerization	Demonstrates context-dependent, antagonist-like behavior of probiotic peptidoglycan at TLR2/1; reveals structural determinants underlying immunological divergence between pathogenic and probiotic peptidoglycan	Waehama et al. (2025)
*Bifidobacterium animalis KL101*	*In vitro*	Anti-osteoclastogenic/anti-inflammatory; regulation of oxidative stress and autophagy	Peptidoglycan suppresses NFATc1 and osteoclast-specific genes (MMP-9, Cathepsin K); reduces ROS, upregulates HO-1, inhibits NF-κB signaling; modulates autophagy (↓ LC3-II/LC3-I ratio, restores p62)	Inhibition of osteoclast differentiation and function; potential therapeutic effect in bone-loss disorders (osteoporosis, periodontitis)	Jee et al. (2025)
*Bacillus pumilus SE5*	*In vivo*	Growth promotion, immune enhancement, intestinal health	Heat-inactivated peptidoglycan and LTA alone or in combination; synergistic activation of NOD2 signaling, upregulation of antimicrobial peptides (epinecidin-1, hepcidin-1, β-defensin), enhanced IgM and complement C3; modulation of pro- and anti-inflammatory cytokines; improved intestinal enzyme activity and microbiota composition	Peptidoglycan +LTA mixture (PL1) yielded optimal growth performance, intestinal health, and immune responses; synergistic effect superior to peptidoglycan or LTA alone	Xu et al. (2025)
*Limosilactobacillus reuteri*	*In vitro* and *in vivo*	Protective/immunomodulatory/antioxidant; mitigates toxin-induced growth retardation, liver damage, and immunosuppression	Peptidoglycan adsorbs AFB1 toxin (64.3–75.9% efficiency *in vitro*); improves antioxidant enzyme activity (glutathione peroxidase), enhances IgA, IgG, IgM, and NDV antibody titers, and reduces liver steatosis	Improved growth performance, feed conversion, immunity, antioxidant status, and liver pathology; optimal dose 200 mg/kg feed	Zhu F. et al. (2024)
*Prohep (probiotic mixture)*	*In vivo;* AOM/DSS-induced colorectal cancer (CRC) murine model	Anti-tumorigenic/anti-inflammatory/microbiota modulation	Suppression of STAT3 phosphorylation, induction of apoptotic p53, modulation of gut microbiota composition (increase in beneficial bacteria, decrease in CRC-associated bacteria); regulation of microbial metabolites, including acetate, and reduction of CRC-associated PG, LPS, and uric acid biosynthesis	Significant reduction in tumor count, tumor size, colonic inflammation, and fibrosis; improved colonic crypt structure; beneficial shifts in gut microbiota and microbial metabolites	Leung et al. (2025)
*Limosilactobacillus fermentum LAC92* (soluble peptidoglycan fragments; SPFs)	*In vitro*	Antiproliferative and pro-apoptotic bioactivity; anticancer potential	Soluble peptidoglycan fragments (SPFs) derived from cell-free supernatant; identification of peptide 92; interaction with MDM2, leading to p53 pathway activation (in silico docking); cytostatic and antiproliferative effects	Significant inhibition of HCT116 proliferation; induction of apoptosis; antimicrobial activity; identification of SPFs as postbiotic anticancer candidates	Fuochi et al. (2023)
*Lactobacillus rhamnosus CRL1505*	*In vivo*	Enhancement of systemic and respiratory innate immunity; mucosal immune restoration	Peptidoglycan and cell wall components stimulate innate immune cells in the respiratory tract; increased recruitment of phagocytes and modulation of cytokine responses (↑ IL-1β, IL-6, TNF-α, ↑ regulatory IL-10)	Complete clearance of pneumococci from lung and blood; restoration of immune responses to levels comparable or superior to well-nourished mice; proof-of-concept that non-viable probiotics and peptidoglycan act as safe immunobiotics	Kolling et al. (2015)
*Levilactobacillus brevis 23,017*	*In vitro, in vivo,* and *ex vivo*	Trained innate immunity; vaccine adjuvanticity; broad-spectrum antimicrobial protection	Peptidoglycan backbone (BLPs) activates TLR2, inducing IL-6–JAK–STAT3 signaling → metabolic reprogramming of macrophages and trained immunity phenotype (↑ cytokine production, ↑ phagocytosis)	Enhanced vaccine efficacy against MRSA; protection even without specific antigen; durable, heterologous innate immune memory	Niu et al. (2025)
*L. casei*, *L. johnsonii*, *L. plantarum*	*In vitro*	Inhibits IL-12 production, regulates inflammation	Peptidoglycans from lactobacilli inhibit IL-12 production induced by *L. casei* via TLR2 and NOD2	Peptidoglycan from *Lactobacillus* strains inhibits IL-12 production, suggesting regulation of gut immunity via recognition receptors	Shida et al. (2009)
General bacterial peptidoglycan (polymeric form)	*In vitro*	Induces robust cytokine production	Polymeric peptidoglycan activates NOD proteins, requiring internalization and lysosomal trafficking for full response	Polymeric peptidoglycan induces proinflammatory cytokines in innate immune cells, unlike monomers such as muramyl dipeptide	Iyer and Coggeshall (2011)
*Bifidobacterium bifidum*	*In vivo*	Macrophage activation and tumor immune response	Whole peptidoglycan induces the production of IL-6, IL-12, TNF-α, and NO, which are involved in antitumor immune reactions	Whole peptidoglycan induces antitumor activity and macrophage-mediated cytotoxic effects	Wang et al. (2001)
*L. casei* CRL431 and *L. paracasei* CNCMI-1518	*In vivo*	Th1-type cell-mediated immunity	Probiotic-derived cell wall interacts with host receptors, inducing Th1-cell polarizing cytokines (increase in IFN-γ/IL-4 ratio)	Reduction of bacterial burden in spleen and liver after *Salmonella* challenge. Augmented delayed-type hypersensitivity response.	Lemme-Dumit et al. (2021)
*Lacticaseibacillus casei* BL23	*In vivo*	Phagocytosis, cytokine secretion, Th1 and Th17 polarization	*L. casei* cell wall integrity (via Lc-p75 enzyme) influences phagocytosis and cytokine secretion. Lc-p75 mutation leads to defective internalization and impaired cytokine secretion.	Reduced Th1 and Th17 cell activation with Lc-p75 mutation and defective phagocytosis; impaired immunomodulatory response.	Tóth et al. (2022)
*Lactobacillus* sp. *peptidoglycan*	*In vitro* and *in vivo*	Th1-dominant immune response	*Lactobacillus* peptidoglycan activates TLR-NF-κB and Jak–STAT pathways in macrophages, inducing inflammatory responses. *In vivo,* this led to Th1 dominance.	Protective inflammatory response with Th1 dominance, anti-colon tumor effect *in vivo.*	Sun et al. (2005)
*L. plantarum*	*In vitro*	Inflammatory cytokine secretion	MyD88-dependent TRAF6/MAPK pathway	Increased secretion of IL-6, IL-1β, and TNF-α; variable immunomodulatory ability of *L. plantarum* dependent on D-Ala-ended precursors.	Song et al. (2021)
*L. acidophilus*	*In vitro*	Inhibition of inflammation in LPS-induced macrophages	Peptidoglycan from *L. acidophilus* inhibits the expression of inducible NO synthase (iNOS) and cyclooxygenase-2 (COX-2) in macrophages.	Significant decrease in iNOS and COX-2 levels, demonstrating anti-inflammatory effects.	Wu et al. (2013)
*Lactobacillus strains*	*In vitro*	Inhibition of pro-inflammatory cytokines (IL-6, TNF-α, IL-1β) in macrophages	Peptidoglycan from different *Lactobacillus* strains interact with TLR-4 signaling, reducing inflammatory cytokine production in macrophages.	Peptidoglycans exhibited structural diversity, yet consistently suppressed LPS-induced inflammatory cytokine release.	Wu et al. (2015)
*B. bifidum*	*In vivo*	Induction of apoptosis in colorectal carcinoma cells, modulation of apoptosis-regulating genes (bcl-2, bax)	Peptidoglycan from *B. bifidum* downregulates *bcl-2* and upregulates *bax* expression, promoting apoptosis of colorectal carcinoma cells.	Increased apoptosis in tumor cells, higher *bax* expression, lower *bcl-2* expression	Li-sheng et al. (1999)
*Lactobacillus bulgaricus*	*In vitro*	Inhibition of cell migration and decreased cell viability in glioblastoma cells	Muramyl pentapeptide (MPP) from *Lactobacillus bulgaricus* decreases cell viability and migration by depleting NADH and upregulating PARP1 and NF-κB.	Decreased cell migration, reduced viability, depletion of NADH, upregulation of PARP1 and NF-κB	Nedzvetsky et al. (2020)
*Lactobacillus paracasei subsp. paracasei M5*	*In vitro*	Cytotoxic effects, apoptosis induction	Whole peptidoglycan from *Lactobacillus paracasei* induces apoptosis in HT-29 cells via mitochondrial-mediated pathways, upregulating proapoptotic genes and downregulating antiapoptotic genes.	Dose-dependent cytotoxicity, apoptosis induction, mitochondrial pathway activation	Wang et al. (2018)
*Lactobacillus paracasei subsp. paracasei X12*	*In vitro*	Immunogenic cell death induction	Integral peptidoglycan (X12-PG) induces immunogenic cell death (ICD) in HT-29 cells by targeting the endoplasmic reticulum (ER), inducing calreticulin translocation, HMGB1 release, and elevated intracellular [Ca2+].	Induction of immunogenic cell death via ER targeting, calcium elevation	Tian et al. (2015)
*L. acidophilus*	*In vitro*	Antitumor activity enhancement	Peptidoglycan from *L. acidophilus* modified by selenylation (Se-Peptidoglycan) enhances antitumor activity by increasing cytotoxic effects in HT-29 cells in a dose-dependent manner.	Se-Peptidoglycan exhibited greater antitumor activity compared to non-modified Peptidoglycan	He et al. (2017)
*Limosilactobacillus fermentum* (LAC92)	*In vitro*	Anticancer activity: antiproliferative, apoptosis induction	Soluble peptidoglycan fragments (SPFs) from *Limosilactobacillus* fermentum induce apoptosis and antiproliferation in HCT116 cells by interacting with MDM2 protein to activate p53.	SPFs exhibited antiproliferative and cytostatic effects, inducing apoptosis in HCT116 cells	Fuochi et al. (2023)
*Lactobacillus reuteri*	*In vitro*	Suppression of periodontitis-associated inflammation	Lr-Peptidoglycan inhibited the production of IL-1β, IL-6, and CCL20 by reducing PI3K/Akt, MAPK, and NF-κB activation through TLR4 suppression.	Lr-Peptidoglycan significantly reduced inflammatory cytokines, with mutanolysin-digested peptidoglycan showing the strongest effects. MDP-like motifs may contribute to anti-inflammatory activity.	Kim et al. (2023)
*L. rhamnosus CRL1505*	*In vivo* and *in vitro*	Modulation of inflammation and coagulation during respiratory viral infections	Peptidoglycan -Lr1505 modulates TLR3-induced inflammation and coagulation responses by increasing IL-10, reducing tissue factor (TF) in F4/80 cells, and normalizing hemostatic parameters in plasma and lungs	Reduced lung tissue damage, balanced inflammation-hemostasis crosstalk, increased IL-10 levels, decreased procoagulant state	Zelaya et al. (2023)
*L. plantarum CRL1506*	*In vivo*	Partial immune modulation	Peptidoglycan from CRL1506 exhibits some immunomodulatory properties but does not fully replicate the effects of PG05.	Some improvement in immune response but not as pronounced as PG05.	Kolling et al. (2018)
*L. rhamnosus CRL1505*	*In vivo*	Enhances antiviral and antibacterial immunity	Peptidoglycan primes respiratory innate immunity via TLR3 activation, increasing lung CD3^+^CD4^+^IFN-γ^+^ and CD3^+^CD4^+^IL-10^+^ T cells and IFN-β^+^ alveolar macrophages.	Protection against RSV and pneumococcal superinfection through increased IFN-γ, IL-10, and IFN-β, leading to reduced bacterial colonization and inflammation.	Clua et al. (2017)
*LcS*	*In vivo*	Anti-inflammatory, inhibition of IL-6 production, suppression of IL-6/STAT3 signaling	PSPG derived from LcS inhibits IL-6 production in LPS-stimulated lamina propria mononuclear cells (LPMCs) by blocking NF-κB activation.	Improved ileitis in the IBD model, inhibition of IL-6/STAT3 signaling, and tumor suppression in the colitis-associated cancer (CAC) model	Matsumoto et al. (2009)
*L. acidophilus (KLDS 1.0738)*	*In vivo*	Anti-allergic activity: modulation of IgE production, Treg/Th17 balance	Peptidoglycan from *L. acidophilus* activates TLR2/NF-κB signaling, influencing cytokine production and improving the Treg/Th17 balance by reducing IgE production and promoting Treg response.	Peptidoglycan modulated allergic inflammation by enhancing Treg response and inhibiting IgE production	Li A. L. et al. (2017)

### 3.1 Innate immunity

Innate immunity represents the first line of host defense and is characterized by rapid, non-specific responses mediated by innate immune cells, such as macrophages and DCs, through PRRs that sense conserved microbial structures (Li and Wu, 2021). Within this context, accumulating evidence supports the hypothesis that *Lactobacilli*-derived peptidoglycan modulates innate immune signaling pathways, particularly by regulating cytokine production. In this sense, accumulating evidence supports the concept that *Lactobacillus*-derived peptidoglycan exerts context-dependent regulation of IL-12 production, rather than a uniform pro- or anti-inflammatory effect. Several studies have demonstrated that purified peptidoglycan from *Lactobacillus* species suppresses macrophage IL-12 production predominantly through TLR2- and NOD2-dependent mechanisms. In particular, Shida et al. (2009) reported that peptidoglycan from *Lactobacillus plantarum*, *Lactobacillus casei*, and *Lactobacillus johnsonii* significantly inhibited IL-12 secretion by macrophages by downregulating *IL-12p40* expression, thereby limiting the formation of bioactive IL-12p70. This inhibitory effect was mediated mainly through TLR2 signaling, with additional contribution from NOD2 recognition of peptidoglycan-derived MDP.

Although peptidoglycan-mediated inhibition was largely dependent on TLR2 signaling, reduced IL-12 production was also observed in TLR2-deficient macrophages, pointing to additional TLR2-independent mechanisms. Watanabe et al. (2004) showed that MDP inhibits IL-12 production via recognition by the intracellular receptor NOD2. In the same way, Shida et al. (2009) noted that L18-MDP, a derivative of MDP, is also likely to be involved in IL-12 suppression. These findings indicate that peptidoglycan inhibits IL-12 production both in its native state through TLR2 and in its processed state (MDP) through NOD2.

In contrast, IL-12 induction reflects distinct experimental conditions, including stimulation with whole bacteria or intracellular digestion-resistant *Lactobacillus* cell wall structures rather than purified peptidoglycan alone (Watanabe et al., 2004; Shida et al., 2009). Whole bacterial cells can engage additional PRRs beyond TLR2, including non-TLR receptors that recognize higher-order cell wall architecture, resulting in enhanced IL-12p35 transcription and apparent Th1 polarization. Importantly, peptidoglycan alone induced markedly lower IL-12 production compared with intact bacteria, even in wild-type macrophages, and this effect was further diminished in TLR2-deficient cells.

Studies investigating *L. casei*–derived peptidoglycan have reported both TLR2/TLR9-dependent and TLR2/TLR9-independent immunomodulatory effects, which may appear inconsistent at first glance (Takeda and Akira, 2005). However, these differences largely reflect variations in experimental context rather than conflicting biological outcomes. TLR2- and TLR9-dependent signaling has been predominantly observed in studies employing intact *L. casei* cells or complex cell wall preparations, where multiple pattern-recognition receptors can be engaged simultaneously at the cell surface or within endosomal compartments. Peptidoglycan, lipoproteins, and Gram-positive bacterial genomic DNA of *Lactobacilli* are reported to activate TLR2 and TLR9, resulting in the release of proinflammatory cytokines (Takeda and Akira, 2005). In contrast, other studies determined that macrophages that were TLR2- or TLR9-deficient or wild-type continue to induce IL-12 when exposed to certain *Lactobacillus* species in the *L. casei* group (Shida et al., 2006; Ichikawa et al., 2007). Unlike peptidoglycan alone, peptidoglycan stimulated low IL-12 in wild-type and even lower in *TLR2*-deficient macrophages (Shida et al., 2009). These results indicate that TLR2 is involved in the impaired IL-12 production caused by peptidoglycan or intracellular digestion-resistant *Lactobacillus* strains. Conversely, yet-to-be-characterized receptors aside from TLRs are likely to recognize the three-dimensional fold of the intracellular digestion-resistant *Lactobacillus* bacterial cell wall and induce augmented IL-12 production. Peptidoglycan-derived fragments are recognized by the NOD1 and NOD2 proteins, which are members of the NLR family of PRRs (Ting et al., 2008; Kawai and Akira, 2010). Both receptors are well-characterized NLRs, with NOD1 recognizing meso-diaminopimelic acid and NOD2 recognizing MDP (Girardin et al., 2003a,b). Though both receptors are highly expressed in immune cells, they are expressed differently in the intestinal epithelium; NOD1 is uniformly expressed in the epithelium, while NOD2 is mainly expressed in Paneth cells in the small intestine (Lala et al., 2003). The PepT1 di/tripeptide transporter, which is highly expressed in the small intestine, is required for the transport of NOD1 and NOD2-activating ligands, such as MDP and tripeptide mesoDAP, into epithelial cells (Dalmasso et al., 2010). NOD1 and NOD2 can also recognize peptidoglycan fragments that have been processed within the phagosome or phagolysosome of antigen-presenting cells (APCs), though translocation transporters for them into the cytoplasm remain to be identified (Philpott and Girardin, 2010).

Fernandez et al. (2011) indicated that peptidoglycan from *Lactobacillus* may augment anti-inflammatory effects through recognition by NOD2. Treatment with Ls33 was found to have protective effects and was linked to enhanced IL-10 production in the colon, a characteristic that is lacking in NOD2-deficient mice. Since NOD2 is a prominent member of the NLR family capable of recognizing peptidoglycan, Fernandez et al. (22) evaluated whether it was involved in the peptidoglycan anti-inflammatory activity of Ls33. A single systemic injection of Ls33 peptidoglycan exacerbated colitis in rats by reducing pro-inflammatory cytokines [TNF-*α*, Interleukin 1 (IL-1)-1β, and Interleukin 6 (IL-6)] with augmented expression of genes involved in the Indoleamine 2,3-dioxygenase (IDO) immunosuppressive pathway in the colon. Although a reduction in *Il10* gene expression was observed in mice treated with *L. salivarius* Ls33–derived peptidoglycan, a localized increase in IL-10 protein levels within the intestinal tissue was consistently associated with the protective effect against colitis (Fernandez et al., 2011). This apparent discrepancy likely reflects the rapid and transient kinetics of *Il10* transcription, coupled with post-transcriptional regulation and localized cytokine secretion at sites of inflammation. Importantly, the enrichment of IL-10 protein in the colonic microenvironment underscores its functional relevance in mediating immune tolerance and tissue protection. Collectively, these findings indicate that probiotic-derived peptidoglycan confers anti-inflammatory effects primarily through localized, IL-10–dependent immunoregulation rather than sustained systemic cytokine expression, highlighting the importance of spatial cytokine dynamics in probiotic-mediated immune modulation.

Treg cells, especially CD4 + CD25 + FoxP3 + subsets, are essential for immune response modulation and tolerance (Coombes et al., 2005). Fernandez et al. (2011) showed that Ls33 peptidoglycan treatment was strongly associated with the increase of CD4 + FoxP3 + Treg cells and CD103 + DCs in mesenteric lymph nodes. Enhanced *Ifng* and *Ido* expression and CD11c + DC enrichment in mesenteric lymph nodes (MLNs) were also found. Although IFN-*γ* is generally associated with pro-inflammatory Th1 responses, increased production in trinitrobenzene sulphonic acid (TNBS)-induced colitis has also been associated with anti-inflammatory activity. In addition, IFN-γ is a major inducer of the IDO-mediated immunosuppressive pathway (Gurtner et al., 2003). Recent research defines the role of the IDO pathway in CD103 + intestinal DCs as the selective proliferation of CD4 + FoxP3 + Tregs (Gurtner et al., 2003). Together, these findings indicate that Ls33 peptidoglycan induces a tolerogenic immune response.

Shida et al. (2009) have already described that peptidoglycan from certain *Lactobacillus* species can suppress macrophage production of IL-12, in favor of the hypothesis that *Lactobacilli*-derived peptidoglycan has functional anti-inflammatory activity. In addition, Watanabe et al. (2004) have shown that MDP activation of NOD2 can suppress TLR2-mediated signaling and Th1 responses. In another study, Watanabe et al. (2008) observed that synthetic MDP cured colitis in mice in a NOD2-dependent fashion. Their finding is in agreement with the hypothesis that NOD2 signaling is the cause of the resolution of inflammation and that NOD2 mutations should worsen IBD by enhancing TLR2-mediated cytokine responses. Interestingly, although it established the binding of NOD2 by Ls33 and NCFM peptidoglycans, as well as by M-tri-N, alone, only Ls33 peptidoglycan had protective activity (Watanabe et al., 2004). New evidence indicates that the transport of NOD2 ligands following the internalization of bacteria and processing in phagolysosomes is facilitated by specific transporters like PEPT1 and PEPT2 (Charrière et al., 2010). It is therefore possible that only the tripeptide M-tri-Lys, a part of Ls33 peptidoglycan, is efficiently transported to the cytosol for recognition by NOD2. While TLR2 activation in peptidoglycan recognition has been disputed, evidence now indicates that peptidoglycan is translocated in a complex with peptidoglycan recognition protein PGLYRP-3 and subsequently activates TLR2-induced phagocytosis (Bu et al., 2010). Phagosome maturation is controlled by TLR through adaptor Myeloid Differentiation Primary Response 88 (MYD88) (Blander, 2007). This can account for the need for MyD88 in protective Ls33 peptidoglycan activity but NOD2 signaling dependency in M-tri-Lys action. The findings indicate that Ls33 exhibits greater peptidoglycan turnover compared to NCFM, which can allow the accumulation and release of M-tri-Lys, hence explaining the greater protective efficacy of Ls33 peptidoglycan (Watanabe et al., 2004). Mechanistically, engagement of TLR2 by *Lactobacillus*-derived peptidoglycan initiates MyD88-dependent signaling that not only regulates cytokine transcription but also promotes cytoskeletal rearrangement and phagocytic uptake of bacterial material (Blander, 2007). Following internalization, peptidoglycan is enzymatically processed within phagolysosomes, leading to the release of defined muropeptides, including M-tri-Lys, which subsequently translocate into the cytosol (Watanabe et al., 2004). These intracellular peptidoglycan fragments are then recognized by NOD2, triggering RIP2-dependent signaling pathways that modulate NF-κB and MAPK activation. Importantly, NOD2 signaling acts as a regulatory checkpoint, attenuating excessive TLR2-driven inflammatory responses while promoting balanced cytokine production, including IL-10 induction.

Huang et al. (2020) confirmed that intact peptidoglycan from *L. rhamnosus* MLGA enhances β-defensin production in a dose-responsive manner in chicken embryo jejunum, ileum, and cecum, and in peripheral blood mononuclear cells (PBMCs), splenocytes, thymocytes, and hepatocytes. Notably, peptidoglycan hydrolysates prepared by lysozyme digestion also stimulated avian β-defensin 9 (AvBD9) induction by PBMCs. Additionally, *L. rhamnosus* MLGA peptidoglycan treatment remarkably enhanced antibacterial activity in PBMC and splenocyte lysates in a dose-responsive manner (Huang et al., 2020). These results indicate that peptidoglycan from certain probiotic bacteria plays a role in host immune modulation and antimicrobial defense through the induction of defensin expression in immune cells and gut tissue. While peptidoglycan fragments also increased AvBD9 expression in PBMCs, their impact was less than the impact of intact peptidoglycan from *L. rhamnosus* MLGA (Huang et al., 2020). In addition, splenocytes also had decreased AvBD9 induction to peptidoglycan hydrolysates, and this could be due to varying levels of PRR expression among immune cell types.

While NOD1 is a cytosolic PRR that senses meso-diaminopimelic acid–containing peptidoglycan motifs predominantly associated with Gram-negative bacteria, NOD2 is the principal intracellular sensor relevant to Gram-positive *Lactobacillus* species, including *L. rhamnosus* MLGA. Huang et al. (2020) demonstrated that peptidoglycan derived from *L. rhamnosus* MLGA enhances innate immune defense by inducing AvBD9 expression in intestinal tissues and peripheral immune cells. This response was accompanied by increased antimicrobial activity, indicating that probiotic-derived peptidoglycan can potentiate host defense mechanisms beyond cytokine modulation (Huang et al., 2020). These findings highlight NOD1 as a functional mediator of probiotic-induced antimicrobial immunity and reinforce the broader role of peptidoglycan fragments in strengthening mucosal and systemic innate immune responses.

Tejada-Simon and Pestka (1999) showed that *L. infantis* peptidoglycan was able to stimulate the RAW 264.7 macrophage cell line to produce TNF-*α* and nitric oxide (NO) at high levels *in vitro*. Consistent with these results, Wang L. S. et al. (2001) showed that the fluorescence intensity of IL-1, IL-6, and TNF-α, and NO content in nude mice peritoneal macrophages were significantly increased following intraperitoneal injection of *B. bifidum* peptidoglycan. These findings show that *B. bifidum* peptidoglycan can efficiently stimulate macrophages to produce large amounts of cytotoxic effector molecules.

Мokrozub et al. (2015) findings showed that the *Lactobacillus delbrueckii sub*sp. *bulgaricus* IMV B-7281 strain with the more elastic cell wall was processed more efficiently by macrophages. The strain was more functionally active, secreted NO, and could accumulate reactive oxygen species than the stiffer cell wall-containing strains *L. casei* IMV B-7280 and *L. acidophilus* IMV B-7279 (Мokrozub et al., 2015). Bacterial substances produced as a consequence of digestion by macrophages seem to be of vital importance in triggering the oxygen-dependent bacteriolytic activity of macrophages and the production of NO against *Lactobacilli*. Interestingly, induction of production of IL-12 was higher in macrophages treated with *L. casei* IMV B-7280 or *L. acidophilus* IMV B-7279, having stronger cell walls compared to *L. delbrueckii* subsp. bulgaricus IMV B-7281 (Мokrozub et al., 2015). Additionally, Мokrozub et al. (2015) found *L. casei* IMV B-7280 was more effective as an inducer of IFN-*γ* production. This indicates that interaction between *Lactobacilli* cell wall constituents and TLR- and/or NOD receptors is the key to inducing cytokine production and activating macrophages. These findings provide proof of the correlation between the plasticity of cell walls in *Bifidobacteria* and LAB and their ability to trigger the macrophage component of the immune response.

### 3.2 Adaptive immunity

Adaptive immunity represents the antigen-specific arm of the immune system, characterized by immunological memory and mediated primarily by T and B lymphocytes, which coordinate cellular and humoral immune responses (Chi et al., 2024). Adaptive immune modulation by probiotics-derived peptidoglycan occurs predominantly through APCs rather than through direct stimulation of lymphocytes. Intracellular sensing of peptidoglycan-derived muramyl dipeptide by NOD2, and to a lesser extent NOD1, requires RIP2 and cooperates with TLR2- and TLR4-mediated signaling under antigen-presenting conditions. While NOD1 recognizes meso-diaminopimelic acid–containing peptidoglycan fragments typically associated with Gram-negative bacteria, NOD2 is the principal intracellular sensor relevant to Gram-positive *Lactobacillus*-derived peptidoglycan. Within this framework, Kobayashi et al. (2005) created *Nod2* knockout mice and observed that the mice had a decreased level of IgG1 production following intraperitoneal immunization with human serum albumin and MDP (Kobayashi et al., 2005). This deficiency was not because of an intrinsic or developmental impairment of the adaptive immune response, since Nod2-deficient mice developed a normal response to TLR7 ligands (Kobayashi et al., 2005). Elaborating on these observations, Magalhaes et al. (2008) showed that activation of NOD2 alone evokes a Th2-type response by inducing Th2-inducing cytokines like MCP-1. NOD2 was also found to play a critical role in the induction of Th1 responses following concurrent stimulation of TLR and NOD2, such as would happen in the scenario of infection (Magalhaes et al., 2008). Disruption of NOD2 or RIP2 signaling impairs this synergistic interaction with TLRs, resulting in defective APC activation and altered cytokine production (Magalhaes et al., 2008). This loss of synergy, rather than isolated *Nod2*deficiency, explains the inability of such mice to mount effective Th1 and Th17 responses following microbial or antigenic stimulation.

Through APC-mediated cytokine modulation, *Lactobacillus*-derived peptidoglycan influences T-helper cell polarization (Fritz et al., 2007). Suppression of IL-12 and IL-23, coupled with enhanced IL-10 production, limits excessive Th1 and Th17 differentiation while preventing pathological inflammation. At the same time, reduced IL-4 availability leads to restrained Th2 skewing, explaining the decrease in IgG1 production, which is primarily dependent on Th2-driven class switching. Fritz et al. (2007) further elucidated the function of Nod1 in the activation of adaptive immune responses. Nod1-deficient mice exhibited abnormalities in the induction of systemic Th1, Th2, Th17, and antibody responses following Complete Freund’s adjuvant (CFA) + ovalbumen immunization. Moreover, intraperitoneal administration of ovalbumen and FK156 (Nod1 ligand) enormously triggered Th2 T-cell activation and IgG1 secretion in a NOD1-dependent process (Fritz et al., 2007). The inability of such mice to develop Th1 and Th17 T-cell immunity was due to interference with synergy with TLR activation. Interestingly, Fritz et al. (2007) demonstrated that *Nod1*-deficient mice’s failure to trigger adaptive immunity was not caused by intrinsic defects in B or T cells. Additionally, they demonstrated that the expression of *Nod1* in non-hematopoietic cells was crucial for the triggering of the Th2 response (Fritz et al., 2007). RIP2, a crucial adaptor molecule downstream of *Nod1* and *Nod2*, is crucial in facilitating adaptive immune responses. *Rip2*-deficient mice developed aberrant cytokine responses to Nod1 and Nod2 ligands, defective macrophage and neutrophil recruitment, and failure to activate Cd11cintCd11b + splenic DCs following stimulation by NOD1 and NOD2 (Magalhaes et al., 2011). *Rip2*-deficient mice also failed to induce a Th2 response to ovalbumen challenges with FK156 or MDP but not with ovalbumen with alum (Fritz et al., 2007). These experiments demonstrate the critical role of peptidoglycan in controlling adaptive immunity. Without TLR ligands, both NOD1 and NOD2 stimulate Th2 responses, indicating that systemic probiotic peptidoglycan release can induce homeostatic Th2 responses (Fritz et al., 2007). Under conditions of bacterial infection, in the presence of both peptidoglycan and TLR ligands, peptidoglycan recognition synergizes with TLR signaling to augment Th1, Th2, and Th17 responses. Although the adaptive immune consequences of NOD1 and NOD2 activation are comparable at the systemic level, their distinct tissue expression patterns make it necessary to further investigate the particular roles of NOD1- and NOD2-regulation of the adaptive immune response in various pathologic settings.

DCs are critical regulators of immunogenic as well as tolerogenic responses based on the nature of encountered antigens, as they can decode the local microenvironment accurately (Coombes and Powrie, 2008). DCs sample directly or indirectly the luminal gastrointestinal tract content, including bacteria and their derived products and metabolites (Coombes and Powrie, 2008). The special ability of DCs to trigger naïve T-cells is attributed to inducing adaptive immunity or tolerance to microbiota constituents (Tóth et al., 2022). Therefore, interaction between LAB, e.g., *Lactobacilli*, and DCs has been the subject of extensive research. In a study, Tóth et al. (2022) analyzed the impact of peptidoglycan alteration in *L. casei* BL23 cell wall on monocyte-derived DCs (moDCs) activation and moDC capacity for T-cell polarization. They demonstrated that the ΔLc-p75 mutant *L. casei* was capable of triggering moDC maturation in parallel with the inhibition of moDC cytokine production (Tóth et al., 2022). Tóth et al. (2022) found that the Lc-p75-deleted *L. casei* mutant would induce weaker Th1 and Th17 responses than the wild-type strain. In contradiction to expectation, peptidoglycans of the wild-type and mutant *L. casei* BL23 strains had a similar immunomodulatory effect on moDCs. It has long been known that Pathogen-associated molecular patterns (PAMPs) are recognized by PRRs on host cells, which will result in the activation of the latter.

Th1-type differentiated cells secrete IFN-*γ*, which is an important cytokine during the initial phase of bacterial infection (Lemme-Dumit et al., 2021). IFN-γ enhances phagocyte-mediated protective immunity and suppresses Th2-type humoral immunity (Pashine et al., 1999; Gordon et al., 2005). Lemme-Dumit et al. (2021) findings showed that whole probiotic bacteria and probiotic cell walls induced similar levels of IFN-γ. This indicates that probiotics are able to stimulate the Th1-type cell-mediated immune response, thereby suppressing the replication and dissemination of enteric pathogens. Therefore, *L. casei* Lc431 treatment elevated the ratios of IFN-γ- and IL-10-secreting cells in the lamina propria and their level in intestinal fluid (Castillo et al., 2013). Moreover, as a response compatible with the Th1-predominant type elicited by cell wall constituents and whole bacteria of the probiotic strain, Lemme-Dumit et al. (2021) noticed inhibition of IL-4 production. These results indicate the immunomodulatory action of sub-cellular fractions of probiotic bacteria, i.e., the probiotic cell wall, which is seen to stimulate the acquired immune system through a Th1-type cell-mediated pathway.

*Lactobacillus* peptidoglycan can potentially affect the cytokine and other immunomodulatory factors secreted by macrophages, which have a pivotal role to play in maintaining Th1 and Th2 cell homeostasis (Sun et al., 2005). One was observed to result in mild inflammation in this research by eliciting a dose of peptidoglycan, whereas three doses of peptidoglycan had the potential to boost antigen presentation as well as T-cell differentiation (Sun et al., 2005). *Lactobacillus* peptidoglycan induced expression of IL-12 with a single dose, but three doses upregulated the expression of IFN-γ, indicating that the immune response might be altered to a Th1-type [cell-mediated immunity (CMI)] response. CMI response is critical in tumor inhibition (Sun et al., 2005). It was also shown here that *Lactobacillus* peptidoglycan could slow down CT26 colon cancer growth by eliciting increased CMI.

Enhanced phagocytic activity, natural killer cell activity, T- and B-cell immune response, and antigen-specific antibody production are responsible for enhanced resistance to tumor growth (Chester et al., 2015). Activation of macrophages is required for all types of tumoricidal activity (Gordon, 2003). PAMPs interact with PRRs on the cell surface, activating macrophages (Chen et al., 2025). Studies have identified that cell fragments of *Lactobacillus murinus* and the latter itself migrate through the wall of the intestine, either through penetration of the epithelial lining or via Peyer’s patches (Plant and Conway, 2001). Resident flora like *Lactobacilli* can pass through the intestinal mucosal barrier and remain in the spleen or other organs for a few days, where they increase phagocytosis activity (Plant and Conway, 2001).

It also observed that varying doses of *Lactobacillus* peptidoglycan influenced the expression of TNF receptor-associated factor 6 (TRAF6) and Toll-interacting protein (Tollip), implying that cytokine expression is regulated by activating or inhibiting these molecules (Sun et al., 2005). The results suggest that the increased activation of TLR-Nuclear factor kappa B (NF-κB) signaling by *Lactobacillus* peptidoglycan is due to enhanced expression of TRAF6 (Sun et al., 2005). While different strains of *Lactobacillus* are commonly applied, they can induce different systemic or mucosal immune responses based on the dose administered (Xin and Zhang, 2002). Maassen et al. (2003) showed that the growth phase of orally given *Lactobacillus* strains can influence the subclass distribution of antigen-specific antibodies like IgG1 and IgG2a. This heterogeneity in antibody response is a result of variations in bacterial cell compositions, which vary with the growth stage (Link-Amster et al., 1994).

Sun et al. (2005) determined that *Lactobacillus* peptidoglycan triggered varied immunological reactions and established that variations in cationic antimicrobial peptide (CAMP) compositions and the quantity delivered to macrophages determine the immune stimulatory activity of *Lactobacilli*. Finally, *Lactobacillus* peptidoglycan-induced immune response is Th1 type and dose-independent (Cross, 2002; Sun et al., 2005). *Lactobacillus* peptidoglycan influences the regulation of regulatory signaling molecules involved in the modulation of cytokine profiles and lymphocyte activation. Pro-Th1 immune signals produced by probiotic *Lactobacilli* at the gut mucosal interface can induce systemic cell-mediated immunity in the context of cancer immunotherapy (Cross, 2002).

### 3.3 Anti-inflammatory capacities

Probiotics have strain-specific anti-inflammatory activities (Bibiloni et al., 2005; Lammers et al., 2005). Anti-inflammatory effects of *Lactobacillus*-derived peptidoglycan are primarily mediated through host innate immune cells, particularly macrophages and DCs, rather than through direct bacterial suppression of cytokines (Katz, 2003; Hanauer and Sparrow, 2004; Kruis et al., 2004). In recent work, Foligne et al. (2007) established the role of NOD2 in *L. rhamnosus* strain Ls33’s protective activities in a murine model of TNBS-induced colitis. Protection correlated with local IL-10 expression. NOD2 is capable of recognizing peptidoglycan-derived muropeptides, including MDP, and this is the reason behind the protective role of Ls33 peptidoglycan, which exhibited anti-inflammatory activity *in vivo* and was determined to activate NOD2 signaling (Foligne et al., 2007). The protective effect that was observed was correlated with a marked decrease in pro-inflammatory genes in the colon. Systemic and oral administration were both capable of inducing this effect, and this indicates that it could be through the induction of regulatory immune responses (Foligne et al., 2007).

Shida et al. (2009) demonstrated that although peptidoglycan stimulation of macrophages can induce transcription of *IL-12p35*, it simultaneously suppresses *IL-12p40* expression, thereby preventing the secretion of biologically active IL-12p70. This regulatory effect was particularly evident for peptidoglycan derived from *L. plantarum* and *L. casei*, supporting the notion that peptidoglycan does not uniformly promote Th1 immunity. Because IL-12p40 is also a shared subunit of Interleukin 23 (IL-23), peptidoglycan-mediated suppression of *IL-12p40* may additionally limit Th17 expansion, providing a mechanistic explanation for the observed anti-inflammatory effects of probiotic peptidoglycan in models of IBD and autoimmunity (Haskó and Szabó, 1999; Peluso et al., 2006). Recent research also points towards the central role of IL-17-producing Th17 cells in inflammation in these diseases (McKenzie et al., 2006; Bettelli et al., 2007). Since IL-12p40 is a component of IL-23, a key cytokine for Th17 cell proliferation and survival, peptidoglycan can potentially suppress these inflammatory disorders by blocking IL-12 and IL-23 production (Shida et al., 2009). Differences in IL-12 outcomes among *Lactobacillus* species can be attributed to strain-specific variations in peptidoglycan composition, intracellular processing, and receptor engagement. Peptidoglycan fragments that efficiently activate NOD2 signaling preferentially suppress TLR-mediated IL-12 production, whereas digestion-resistant cell wall structures derived from intact bacteria may promote IL-12 induction through alternative receptor pathways. These findings collectively indicate that probiotic peptidoglycan functions as a fine-tuner of cytokine responses rather than a fixed inducer of inflammation.

Excess production of iNOS may result in the generation of excessive amounts of NO, which is toxic to inflammatory cells (Song et al., 2021). Compared with the wild-type *L. plantarum* AR113 strain, the overexpression-modified strains showed significantly increased expression of iNOS. The incorporation of D-Ala-ended peptidoglycan into the bacterial cell wall led to elevated *iNOS* gene expression (Song et al., 2021). The cell wall of the *L. plantarum* even has immunologic stimulating potential by becoming bound with the TLR2 and the other macrophage receptors to induce cell signaling pathways and evoke downstream immunity. Although it had been found to stimulate TLR2, TLR4, and the MAPK pathway through D-Ala, its potential to support TRIF and the IKK (IκB Kinase) pathways was not present. This indicates that strains bound to TLR2 and TLR4 on the surface of macrophages entered cells and stimulated the MyD88-dependent pathway (Song et al., 2021). Of strains tested, *L. plantarum* AR113△ldhL△ldhD, with the highest ability to bind D-Ala-ended peptidoglycan precursors, showed the highest *iNOS* expression, more than fourfold higher than that of wild-type *L. plantarum* AR113. The pathway-specific proteins examined via polymerase chain reaction (PCR) for the macrophages confirmed that only TLR2 and TLR4 showed expressed DNA bands, thus proving the activation of the said two pathways. The findings substantiated that the four strains (AR113, AR113-DdlLc, AR113△ldhL△ldhD, and AR113△ldhL△ldhD-DdlLc) activated TLR2 and TLR4 to induce activation of the TRAF6/MAPK pathway while suppressing the activation of the TRIF and IKK pathways (Song et al., 2021). The DdlLc overexpression raised the level of D-Ala at peptidoglycan ends, markedly enhancing macrophage proinflammatory cytokine release in co-culture (Song et al., 2021). The previous research showed that D-Ala removal from LTA branch chains of *L. plantarum* caused some anti-inflammatory effects, which indicates that D-Ala was pro-inflammatory (Song et al., 2021).

Recent advances in peptidoglycan profiling, enabled by the peptidoglycan _MS2 *in silico* MS/MS library, have revealed a remarkable diversity of peptidoglycan chemotypes in gut bacteria and uncovered bioactive peptidoglycan forms with anti-inflammatory properties (Kwan et al., 2024). Through these efforts, an unexpectedly high abundance of anhydro-N-acetylmuramic acid (anhydro-NAM)-bearing peptidoglycan produced through the actions of lytic transglycosylase (LT) enzymes was discovered among *Bifidobacterium* bacteria, which comprise model probiotic microorganisms (Kwan et al., 2024). In functional terms, these anhydro-peptidoglycans have strong anti-inflammatory effects. Evidence has emerged to suggest that pre-treatment with *Bifidobacterium*-derived anhydro-peptidoglycan can strongly inhibit pro-inflammatory cytokines in response to LPS in murine macrophages (Kwan et al., 2024). Biochemical analyses revealed the presence of three enzymes in *Bifidobacterium* that produce LT, named MltG, RpfB-FL, and RpfB-truncated, which possess complementary activities that together account for the abundance of anhydro-peptidoglycan (Kwan et al., 2024). This structural adaptation underlines the particular function by which probiotic bacteria produce peptidoglycan fragments that promote immune tolerance over immune activation. In summary, these findings show that anhydro-peptidoglycans derived from probiotic bacteria play an important role in maintaining immune homeostasis by suppressing excessive immune activation.

## 4 Probiotic-derived peptidoglycan and signaling pathways

Probiotic-derived peptidoglycan serves as a critical microbial cell wall component that interacts with the host immune system through signaling pathways. Interestingly, peptidoglycan derived from the probiotic strain BBMN68 exhibits a selective immunomodulatory role (Zhou et al., 2025). *In vitro* studies demonstrated that BBMN68-derived peptidoglycan inhibited TNF-*α* and IL-1β expression in LPS-stimulated macrophages, suggesting that probiotic peptidoglycan can attenuate excessive inflammation while supporting immune homeostasis (Zhou et al., 2025). This contrasts with uncontrolled peptidoglycan-mediated activation, which typically promotes inflammation. Furthermore, other bacterial cell wall components—such as lipoteichoic acid (LTA), wall teichoic acid (WTA), and surface-layer proteins (SLPs)—may synergize with peptidoglycan to enhance immunomodulatory effects (Zhou et al., 2025).

Peptidoglycan serves as an important postbiotic molecule derived from probiotics such as *Bifidobacterium animalis sub*sp. *lactis KL101* (Jee et al., 2025). It has been shown to influence osteoclast differentiation and activity by modulating several intracellular signaling pathways triggered by Receptor activator of NF-κB ligand (RANKL), the key cytokine responsible for osteoclastogenesis. Peptidoglycan interferes with the activation of Nuclear Factor of Activated T-cells, cytoplasmic, calcineurin-dependent 1 (NFATc1), a central transcription factor driving osteoclast formation, thereby reducing the expression of downstream osteoclast-specific genes such as Tartrate-resistant acid phosphatase (TRAP), Matrix metalloproteinase-9 (MMP-9), and Cathepsin K (Jee et al., 2025). These genes are critical for bone resorption, and their suppression by peptidoglycan limits osteoclast maturation and functional activity. In addition, peptidoglycan mitigates intracellular oxidative stress by lowering reactive oxygen species (ROS) levels, which are normally elevated during RANKL-induced osteoclastogenesis. This reduction in ROS dampens NF-κB activation and nuclear translocation, suppressing the transcription of genes essential for osteoclast differentiation and breaking the positive feedback loop that promotes bone resorption (Jee et al., 2025). Peptidoglycan also enhances the cellular antioxidant response by upregulating heme oxygenase-1 (HO-1), an enzyme that counteracts oxidative stress and further inhibits NF-κB signaling. Through this dual effect on ROS reduction and HO-1 induction, peptidoglycan provides a protective environment that limits osteoclast development. Finally, peptidoglycan modulates autophagy in osteoclast precursors by decreasing the LC3-II/LC3-I ratio and increasing p62 levels, thereby restraining excessive autophagic activity that supports osteoclast survival and bone resorption (Jee et al., 2025). Together, these mechanisms reveal that KL101-derived peptidoglycan exerts a multi-targeted inhibitory effect on osteoclastogenesis.

In the context of colorectal cancer (CRC), excessive peptidoglycan translocation is known to trigger macrophage activation, IL-6 secretion, and subsequent Signal transducer and activator of transcription 3 (STAT3) -mediated inflammation, promoting tumor proliferation and fibrosis (Leung et al., 2025). In the current study, Prohep downregulated peptidoglycan biosynthesis, thereby limiting the pro-inflammatory cascade that would otherwise exacerbate CRC progression. This suppression contributed to reduced activation of STAT3, a transcription factor linking inflammation to tumor growth, while simultaneously enhancing apoptotic signaling via p53 (Leung et al., 2025). The effect was a reduction in tumor number and size, improvement of colonic architecture, and mitigation of collagen fibrosis. Moreover, Prohep’s modulation of peptidoglycan -associated pathways intersected with gut microbiota dynamics. By promoting beneficial bacteria such as *Desulfovibrio porci* and *Prevotella sp.* PTAC, Prohep may have further reduced pro-tumorigenic peptidoglycan -mediated inflammatory signaling (Leung et al., 2025). Collectively, these findings suggest that probiotic-derived peptidoglycan signaling acts as a key mediator linking gut microbiota modulation to anti-inflammatory and anti-tumor responses. By suppressing harmful peptidoglycan production while promoting beneficial microbial metabolites, Prohep effectively counteracts CRC progression through peptidoglycan -mediated immune and metabolic pathways.

Additionally, peptidoglycan-derived soluble polysaccharides (PSPGs) derived from *L. casei Shirota* (LcS), and specifically its component PSPG-I, have been shown to selectively inhibit IL-6 production in LPS-stimulated lamina propria mononuclear cells (LPMCs) and macrophages, without affecting other strains of *Lactobacilli* (Matsumoto et al., 2009). This action is achieved through downregulation of NF-κB phosphorylation, which prevents excessive inflammatory signaling, including the IL-6/STAT3 axis that is central to both IBD and colitis-associated cancer (CAC) (Matsumoto et al., 2009).

The anti-inflammatory effect of PSPG-I is independent of its internalization by immune cells and can be blocked by specific monoclonal antibodies against PSPG-I, highlighting its direct interaction with immune cell surface components. In addition, LcS treatment induces Nod2 expression in macrophages, a receptor that recognizes MDP in peptidoglycan and mediates negative regulation of pro-inflammatory signaling, thereby limiting NF-κB activation and cytokine production (Matsumoto et al., 2009). This Nod2-mediated pathway is especially relevant in preventing IBD and CAC, as defective NOD2 signaling is associated with increased susceptibility to intestinal inflammation. The immunomodulatory effects of probiotic-derived peptidoglycan are further enhanced by its stability within macrophages; LcS is relatively resistant to intracellular digestion, enabling sustained activation of NOD2 and downstream anti-inflammatory responses (Matsumoto et al., 2009). These findings collectively suggest that probiotic peptidoglycan functions as a key signaling molecule, linking bacterial cell wall components to host immune regulation by selectively modulating NF-κB and IL-6/STAT3 signaling pathways, thereby contributing to both intestinal homeostasis and inflammation suppression. Collectively, these findings indicate that probiotic-derived peptidoglycan functions as a key mediator of host-microbe signaling, balancing immune activation and regulation to maintain intestinal and systemic immune homeostasis.

## 5 Application of probiotics-derived peptidoglycan on selected diseases

**Figure 2:**
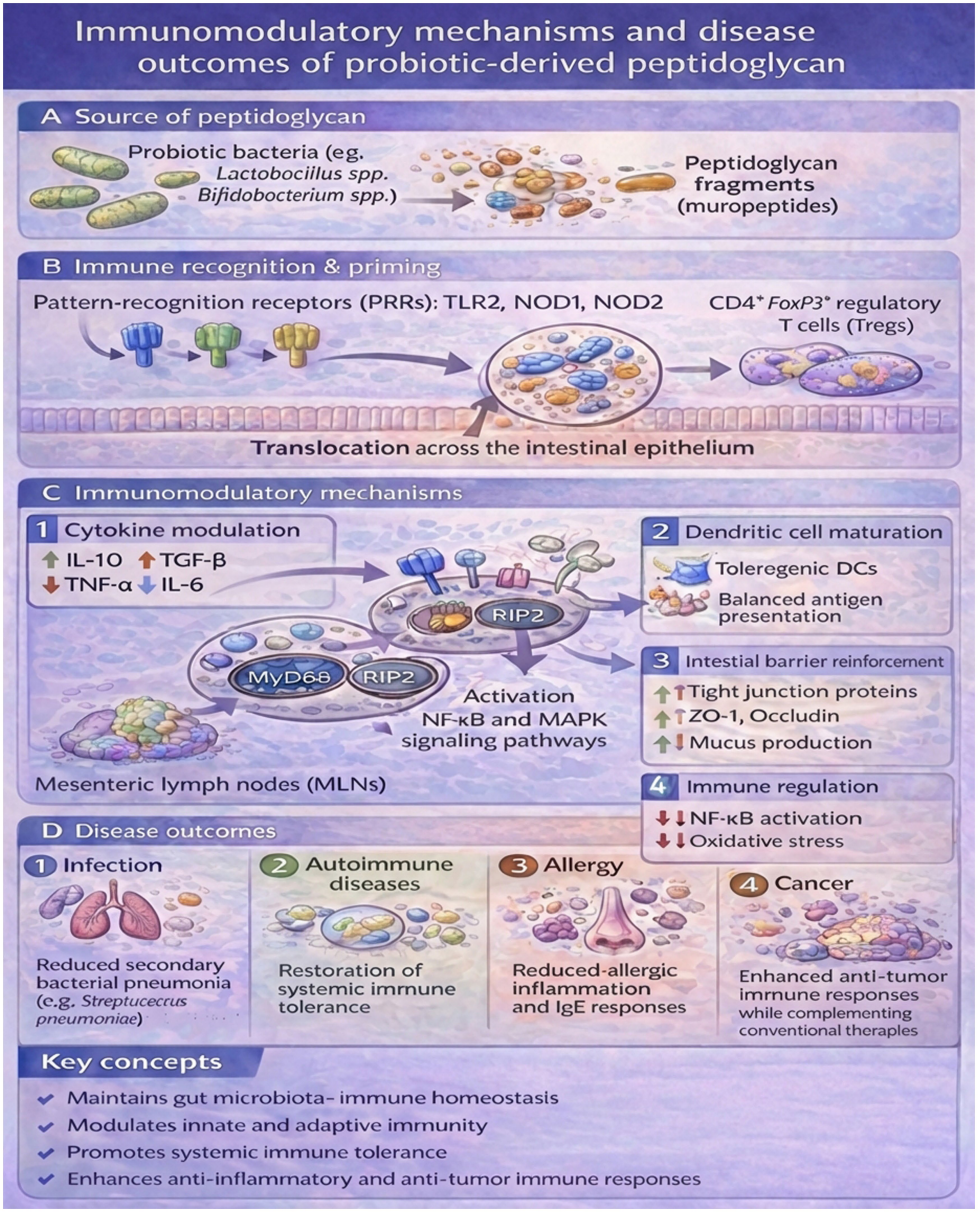
Immunomodulatory mechanisms and disease outcomes of probiotic-derived peptidoglycan (PG). **(A)** Probiotic bacteria (e.g., *Lactobacillus spp*. and *Bifidobacterium spp.*) release peptidoglycan fragments (muropeptides) in the intestinal lumen. **(B)** PG fragments cross the intestinal epithelium and are recognized by pattern-recognition receptors (PRRs), including TLR2 (MyD88-dependent) and NOD1/NOD2 (RIP2-dependent), activating NF-κB and MAPK signaling and promoting immune priming in mesenteric lymph nodes (MLNs) with induction of CD4^+^FoxP3^+^ regulatory T cells (Tregs). **(C)** Downstream immunomodulatory mechanisms include: (C1) cytokine modulation (↑IL-10, ↑TGF-β, ↓TNF-α, ↓IL-6); (C2) tolerogenic dendritic cell maturation and balanced antigen presentation; (C3) reinforcement of the intestinal barrier via increased tight-junction proteins (e.g., ZO-1, occludin) and mucus production; (C4) immune regulation through Treg induction and Th1/Th17 balance and inflammation control via modulation of NF-κB signaling and oxidative stress. **(D)** These effects are associated with improved disease outcomes, including (D1) reduced secondary bacterial pneumonia caused by Streptococcus pneumoniae following viral respiratory infection, (D2) restoration of systemic immune tolerance in autoimmune disorders, (D3) attenuation of allergic inflammation, and (D4) enhanced anti-tumor immune responses.

Due to the failure of conventional medicine in controlling disorders associated with gut microbiota, coupled with the growing demand for efficient disease control and the improvement in biomedical technologies, there has been mounting demand for new therapeutic approaches (Lynch et al., 2019). Both probiotics and probiotic peptidoglycan have shown promise as complementary or alternative therapies, being capable of improving health and slowing disease by normalizing gut microbiome balance (Tegegne and Kebede, 2022). Probiotics-derived peptidoglycan functions primarily as an immunomodulatory effector molecule, rather than as a direct modifier of the microbial ecosystem. Local treatments have succeeded in curing gastrointestinal diseases, such as IBD, antibiotic-associated diarrhea, and colorectal cancer (Figure 2) (Varsha et al., 2021).

### 5.1 Infection

Studies involving *L. rhamnosus* indicate that its protective effects during infection are primarily mediated through modulation of host inflammatory responses rather than direct reduction of pathogen burden. Peptidoglycan represents one of several bioactive bacterial components capable of shaping innate and adaptive immunity, acting in concert with other cell wall–associated molecules (Kolling et al., 2015). Kolling et al. (2015) findings showed that *L. rhamnosus* CRL1505 peptidoglycan can be used as an effective mucosal immunomodulator for increasing protection against respiratory infections. Additionally, identification of the mechanisms by which *L. rhamnosus* CRL1505’s peptidoglycan mechanisms of action can assist in the rational design of effective and safe therapies to reinforce respiratory immunity in immunocompromised patients (Kolling et al., 2015).

Like active *L. rhamnosus* CRL1505, its purified peptidoglycan (PG1505) intake before nasal infection with poly(I: C) strongly increased the production of type I IFNs, especially IFN-*β*, in bronchoalveolar lavage (BAL) (Clua et al., 2017). In turn, PG1505 enhanced the expression of several interferon-stimulated genes that suppress viral replication. Moreover, PG1505 also regulated production of pro-and anti-inflammatory cytokines in response to TLR3 stimulation, the cytokines whose production is pivotal in establishing an effective antiviral defense (Clua et al., 2017). PG1505 enhanced the production of certain cytokines (TNF-*α*, IL-10) but reduced the production of others (IL-6, MIP-1), indicating it regulates inflammation. PG1505 treatment enhanced the capacity to regulate respiratory syncytial virus (RSV) replication in the lung and inflammatory damage in lung tissue (Clua et al., 2017). The effect was mimicked by viable *L. rhamnosus* CRL1505, because PG1505 enhanced the frequencies of CD3 + CD4 + IFN-*γ*+, MHCII+CD11b^lowCD103+, and MHC-II + CD11b^highCD103- DCs, as well as augmented the respiratory levels of IFN-γ to indicate the induction of a Th1 response, which is a critical mechanism of immunity against viral infection of the respiratory tract (Clua et al., 2017). The findings point to peptidoglycan as one of the significant components of bacteria responsible for antiviral and immunomodulatory activities of *L. rhamnosus* CRL1505.

Notably, in some infection models, improved survival occurs despite only modest or insignificant reductions in pathogen load. This suggests that protection is achieved through enhanced immune regulation and limitation of immunopathology, rather than pathogen elimination alone. Increased production of IFN-γ likely supports effective antimicrobial immune activation, while concurrent elevation of IL-10 serves to restrain excessive inflammation and tissue damage. One of the most important findings to emerge from the current study is that heat-killed (HK1505) and PG1505 differentially modulated the behavior of infant mice towards secondary pneumococcal pneumonia induced by poly(I: C) or by RSV exposure (Clua et al., 2017). Intranasal administration of the live *Lactococcus lactis* NZ9000 into adult and neonatal mice led to the reduction by approximately 2 logs of cell counts of *Streptococcus pneumoniae* from the lungs (Medina et al., 2008). Also, non-viable *L. casei* CRL431, non-viable *L. rhamnosus* CRL1505, or their peptidoglycan permitted immunocompromised, undernourished mice to completely eradicate *S. pneumoniae* from the lungs (Villena et al., 2009; Kolling et al., 2015).

Although small on respiratory pathogen burden, PG1505 treatment had a dramatic impact on lung tissue damage and dissemination to the blood. These findings are important, as human studies and animal models of RSV-*S. pneumoniae* superinfection revealed that increased lung injury and enhanced bacteremia are defining factors in regulating infection severity and mortality rates (Hament et al., 2005; Weinberger et al., 2013; Smith et al., 2014; Cebey-López et al., 2016). Infant mice immunized with PG1505 showed better survival after superinfection with *S. pneumoniae* and RSV. RSV superinfection by *S. pneumoniae* stimulated vigorous inflammation, with enhanced accumulation of infiltrating neutrophils and heightened tissue levels of pro-inflammatory mediators in the lung, relative to mice infected with either pathogen alone (Stark et al., 2006).

Clua et al. (2017) found a minimum of three cytokines—IL-10, IFN-β, and IFN-γ—in playing a part in the HK1505- and PG1505-mediated immunomodulatory protective effect of respiratory superinfection. Precisely, IL-10 plays a protective role against inflammation-induced damage, and IFN-γ plays a role in the repression of pneumococcal multiplication in the lungs. These findings are in agreement with a previous study, where it was shown that the protection of infant mice from poly(I: C) or RSV infection by live *L. rhamnosus* CRL1505 was IL-10 and IFN-γ-dependent (Chiba et al., 2013). In addition, HK1505 and PG1505 also augmented the populations of CD3 + CD4 + IFN-γ + and CD3 + CD4 + IL-10 + T cells in baby mice’s lungs, again higher than controls after pneumococcal infection, indicating their protection against rechallenge bacterial pneumonia (Clua et al., 2017).

Kolling et al. (2018) investigated the impact of *L. rhamnosus* peptidoglycan treatment on the numbers and function of effector cells and adaptive immune system mediators for respiratory infections. Malnutrition can significantly alter the bone marrow, thymus, spleen, and lung pools of T cells (Kolling et al., 2018). T-cell reconstitution is required for host defense because they are the ones that produce cytokines, which coordinate and control different mechanisms of immunity and control B-cell antibody production. In Kolling et al. (2018) study, BCD + PG05-treated mice had higher counts of double-negative CD25 + and double-positive T cells following infection, which reflects active thymopoiesis. Additionally, PG05 treatment enhanced the maturation of CD4 T cells in the thymus after pneumococcal challenge, as well as increased the CD3 + CD4 + population in the spleen and sustained high levels of CD3 + CD4 + in the lungs relative to other groups. These treatments, including live *L. rhamnosus* CRL1505, could play an essential role in protection against *S. pneumoniae* infection (Kolling et al., 2018).

PG05 therapy itself might influence cytokine response after respiratory challenge with *S. pneumoniae*. There was marked upregulation of the Th2 cytokine IL-4 in the BAL and serum of undernourished mice treated with BCD + PG05 after pneumococcal infection relative to PG06 or PG534 treatments. This result is consistent with high levels of Immunoglobulin (Ig) A, IgG, and IgM anti-pneumococcal antibodies in the BAL and serum of the BCD + PG05 group, as in other studies (Kolling et al., 2018). Moreover, PG05 treatment induced the greatest response of IL-10 in the BAL after infection, which can be linked to less tissue damage and neutrophilic invasion of the airways in this group (Kolling et al., 2018). In addition, only PG05 caused the secretion of IL-2, which is a key cytokine in CD4 + T-cell response to pneumococcal antigens (Kolling et al., 2018). Malnutrition greatly suppresses BAL and serum anti-pneumococcal antibodies and blunts the humoral immunity of malnourished hosts. Also, the opsonophagocytic activity of IgG antibodies is strongly compromised in starved mice, showing failure in the B cell population within bone marrow, while their capacity to form antibodies is unaffected (Kolling et al., 2018). Kolling et al. (2018) confirmed that PG05-treated malnourished mice exhibited a restored immune response to respiratory disease. The BCD + PG05 group also presented with more immature and mature B cells in the lungs and bone marrow, and mature B lymphocytes in the spleen. These are consistent with the elevated local and systemic anti-pneumococcal antibody production in the BCD + PG05 group (Kolling et al., 2018). Peptidoglycan from the probiotic *L. rhamnosus* CRL1505 induced an increase in mature B cells in the lungs, which was reflected in increased IgG in the BAL. BCD + PG534 therapy did not increase the number or function of the B cells, as shown by these findings. This experiment shows that nutritional supplementation of malnourished mice by nasal administration of peptidoglycan of *L. rhamnosus* CRL1505 increases innate immunity and respiratory and systemic adaptive immunity (Kolling et al., 2018).

The worldwide emergence of multidrug-resistant (MDR) bacteria is an important concern for modern healthcare, especially in view of severe coinfections like methicillin-resistant *Staphylococcus aureus* (MRSA), and this significantly fuels morbidity and mortality (Birlutiu and Birlutiu, 2025). Hence, new approaches using the immune system rather than antibiotics are needed urgently. In this sense, probiotic-derived peptidoglycan, specifically in the form of bacterial-like particles derived from LAB, offered great promise as an immune-modulating approach that can be used for the stimulation of immunity against infection through the concept of trained immunity. Trained immunity is defined as the ability of innate immune cells to enter into a memory-like state after the primary exposure, which leads to an accelerated and robust response to subsequent infections with different pathogens. Unlike traditional adaptive immunity, trained immunity is mediated by macrophages, monocytes, and other innate immune cells due to epigenetic and metabolic reprogramming. Recently available evidence suggests that the peptidoglycan backbone of probiotic bacteria (BLP) is an efficient inducer of trained immunity, offering broad-spectrum protection against bacterial infections, including *S. aureus*, in the absence of distinct antigens (Niu et al., 2025). Mechanistically, the peptidoglycan fraction of probiotics is mediated mainly through the pattern recognition receptor TLR2, which is prominently expressed on cells of the innate immune system. Signaling downstream of TLR2 receptor engagement by BLP is mediated through several pathways, including JAK-STAT3, mammalian target of rapamycin (mTOR), and hypoxia inducible factor 1 subunit alpha (HIF-1α), which work in concert to coordinate the metabolic and functional repolarization of macrophages (Niu et al., 2025). Stimulation of these pathways enhances the transition to aerobic glycolysis, as is characteristic of trained immunity, as well as macrophage effector responses such as cytokine secretion and phagocytosis. Transcriptomic studies also showed that genes encoding inflammation, antigen presentation, metabolism, and epigenetic functions are all induced by BLP-stimulated cells to maintain the trained state (Niu et al., 2025). Besides its independent protective roles, there is immense translational potential for the probiotic-derived component, peptidoglycan, as an immunopotentiator and vaccine carrier. With subunit and multi-epitope vaccines, the BLP exerts a profound protective effect on MRSA infection by potently accelerating the activation of the innate immune system and the subsequent establishment of the adaptive immune response (Niu et al., 2025). Compared to conventional adjuvants, there exist numerous benefits in the use of BLPs as vaccine adjuvants. For example, these adjuvants possess excellent safety and stability properties and can be easily produced. Furthermore, they can act as antigen-delivery systems using surface display methods. Significantly, probiotic-derived peptidoglycan-based therapeutic approaches are quite in line with trained immunity-based vaccines that can serve to provide early, overall protection against various pathogens without relying on antibiotics. To some extent, BLPs can serve to provide an added or preventive modality against infections by making it possible to resist secondary infections by various pathogens. In summary, peptidoglycan derived from probiotics is a strong innate immune modulator that can generate trained immunity via a TLR2-mediated pathway, with the capacity to provide heterologous protection and function independently of specific antigens, making it a highly attractive tool for the prevention and treatment of MDR bacterial infections. Future investigation into this substance will, therefore, be critical for optimizing and harnessing this immune modulator into a productive tool for use in managing bacterial infections.

### 5.2 Autoimmune diseases

Earlier research has indicated that the probiotic *L. casei* strain LcS is capable of suppressing various autoimmune disease models in mice, such as arthritis, type I diabetes, murine lupus, and chronic IBD (Matsumoto et al., 2009). In a study, Matsumoto et al. (2009) discovered that LcS and its whole cell wall fraction suppressed IL-6 production in LPS-stimulated LPMCs of mice with chronic IBD. They initially determined the inhibitory activity of various PSPGs of LcS and other *Lactobacillus* species against IL-6 production in LPS-stimulated LPMCs from IBD mice. Matsumoto et al. (2009) validated that PSPG from LcS strongly inhibited IL-6 production. The PSPG in LcS is composed of two fractions: PSPG-I and PSPG-II. Their findings strongly suggest that PSPG-I is responsible for the anti-IL-6 activity of LcS. Purified PSPG-I repressed IL-6 production in LPS-activated RAW cells by repressing NF-kB phosphorylation. Additionally, Matsumoto et al. (2009) revealed that PSPG-I repressed IjB kinase phosphorylation in LPS-activated RAW cells. These findings indicate that PSPG-I represses IL-6 production in inflammatory macrophages, and PSPG-I-positive *L. casei* strain inhibited the induction of IBD and CAC effectively. Earlier studies have indicated that TLR2 and TLR4 are not implicated in LcS anti-IL-6 activity (Matsumoto et al., 2005). Matsumoto et al. (2009) also noted strong induction of Nod2 in RAW cells exposed to LcS. NOD2 activation by MDP suppressed pro-inflammatory cytokine production following TLR2 and TLR4 ligands dramatically (Hedl et al., 2007). These findings highlight the importance of PSPG-I and Nod2 stimulation in mediating the anti-inflammatory activities of *L. casei* LcS, and beneficially guide its application as a therapeutic agent in inflammatory disease.

Fernandez et al. (2011) found the therapeutic application of *Lactobacillus* strain Ls33 GN in producing anti-inflammatory activities through the activation of NOD2 in IBD. Intraperitoneal administration of Ls33 peptidoglycan caused a significant elevation of IL-10 in the colon, which failed to occur in *Nod2*-deficient mice, and points towards the involvement of NOD2 as a mediator of these protective functions (Fernandez et al., 2011). A single systemic injection of Ls33 peptidoglycan exerted a strong suppressive effect on colitis, along with decreases in key inflammatory cytokines (TNF*α*, IL-1β, and IL-6) and overexpression of IDO immunosuppressive pathway-related genes in the colon (Fernandez et al., 2011). In spite of reduced *Il10* gene expression in Ls33 peptidoglycan -treated mice, localized augmentation of IL-10 protein was directly responsible for the protective effect of peptidoglycan. The inconsistency is most probably caused by the fast kinetics of *Il10* gene expression after peptidoglycan treatment. Notably, protection was completely abolished in IL-10-deficient mice, confirming IL-10 as the master regulator of the anti-inflammatory response (Fernandez et al., 2011). Tregs, CD4 + CD25 + FoxP3 + cells, are essential for immune homeostasis and tolerance. In their research, the protective roles of Ls33 peptidoglycan were correlated with enhanced CD4 + FoxP3 + Tregs and CD103 + DCs in the MLNs of peptidoglycan-treated mice. Elevated levels of IFN-*γ* and IDO were also detected in CD11c + DCs in the MLNs (Fernandez et al., 2011). While IFN-γ is most often associated with pro-inflammatory Th1 responses, it can also be anti-inflammatory in some situations, e.g., TNBS-induced colitis. IFN-γ is a key inducer of the IDO-dependent immunosuppressive cascade, which enhances CD4 + FoxP3 + Treg proliferation, especially by CD103 + intestinal DCs (Fernandez et al., 2011).

One of the primary ways by which probiotic-derived peptidoglycan protects against colitis involves stimulating the signaling pathway of NOD2. The NOD2 recognizes the MDP structural component of peptidoglycan (Gao et al., 2022). The association of genetic polymorphisms of NOD2 with Crohn’s disease underscores its significance in the etiology of intestinal inflammation. Besides its significance in human genetics, evidence suggests that the lack of microbiota-derived MDP or NOD2 signaling plays a critical role in the development of colitis (Gao et al., 2022). In this regard, Gao et al. (2022) found that LPH, a probiotic-derived bifunctional peptidoglycan hydrolase with both N-acetyl-β-D-muramidase and D, L-endopeptidase activities, is required for the efficient generation of MDP from various bacterial peptidoglycans. By strongly triggering NOD2 signaling, LPH inhibits inappropriate inflammation and improves epithelial barrier function and immune tolerance in the gut. Moreover, a diminished expression level of DL-endopeptidase genes was found in patients with Crohn’s disease; supplementation with the enzymes corrected the deficiency and protected against DSS-driven colitis in mouse models (Gao et al., 2022). From a functional standpoint, activation of NOD2 by peptidoglycan has regulatory effects on biological processes related to colitis, such as immune conditioning, inhibition of pro-inflammatory cytokines, autophagy, and epithelial regeneration (Gao et al., 2022). These biological processes indirectly modulate colitis by reducing mucosal and intestinal inflammation.

In a DSS-induced murine colitis model by Li et al. (2025), dietary supplementation with peptidoglycan alleviated key disease symptoms, including weight loss, colon shortening, and elevated disease activity index (DAI) scores. Histopathological analyses revealed improved epithelial barrier integrity, restored crypt morphology, and reduced immune cell infiltration, indicating a direct protective effect on intestinal tissue. The anti-inflammatory effects of peptidoglycan are mediated, in part, through modulation of pro-inflammatory cytokines. Peptidoglycan intervention reduced circulating levels of IL-6, IL-1*β*, and TNF-α, reflecting suppression of the inflammatory cascade driven by APCs and macrophages (Li et al., 2025). Mechanistically, peptidoglycan likely interacts with PRRs on immune cells, modulating downstream signaling pathways such as NF-κB, thereby limiting excessive cytokine production while maintaining immune homeostasis. Furthermore, in Li et al. (2025) study, treatment with peptidoglycan enhanced microbial richness and diversity, increased the abundance of beneficial taxa, including *Lactobacillus*, *Lachnospiraceae*, *Dubosiella*, and *Clostridia*, and restored the *Firmicutes*/*Bacteroidetes* ratio. These microbial shifts promoted the production of short-chain fatty acids (SCFAs), particularly propionate, butyrate, and pentanoic acid, which are critical for intestinal barrier integrity, Treg cell differentiation, and suppression of pro-inflammatory responses (Li et al., 2025).

In addition, Ls33 peptidoglycan treatment triggered IL-10-secreting partially mature DCs, which, through adoptive transfer, reduced colitis in mice (Fernandez et al., 2011). The protective effect was, however, inhibited when DCs were isolated from *Nod2* knockout animals, proving the absolute reliance on NOD2 signaling for Ls33 peptidoglycan-induced tolerogenic effects by activating regulatory DCs (Fernandez et al., 2011). These results were in agreement with earlier research, such as that of Shida et al. (2009), which had established that peptidoglycan from some *Lactobacilli* was able to suppress the secretion of IL-12 by macrophages, and Watanabe et al. (2004) who had established that stimulation of NOD2 by MDP was able to suppress TLR2 signaling and Th1-type inflammation. Fernandez et al. (2011) results also support the hypothesis that NOD2 signaling is important to counteract inflammation, and NOD2 mutations can induce overactive IBD by facilitating abnormal TLR2-dependent cytokine release. In conclusion, these results indicate that the peptidoglycan of certain *Lactobacillus* strains, e.g., Ls33, is capable of inducing tolerogenic immune responses via NOD2 signaling and, therefore, is a promising candidate for identifying therapeutic approaches to IBD. Structural and turnover studies of LAB peptidoglycan, including the creation of isogenic mutant pairs, may be useful for obtaining valuable information and selecting the most appropriate strains for potential prophylactic or therapeutic use in the treatment of IBD (Fernandez et al., 2011).

### 5.3 Allergy

Probiotics also contribute to the control of allergic disorders through immunological mechanisms, including enhancement of mucosal IgA production and modulation of allergen-specific T- and B-cell responses (Eslami et al., 2020). These processes are highly intricate and involve coordinated regulation of multiple genes, PRRs such as TLRs, intracellular signaling pathways, and strengthened IgA-mediated immune activity within the intestinal mucosa (Abreu et al., 2005; Toh et al., 2012). Evidence from numerous *in vivo* and *in vitro* investigations, together with findings from human clinical studies, supports a beneficial role for probiotics in allergic conditions. In particular, randomized controlled trials have demonstrated that administration of *L. rhamnosus GG* to expectant mothers with a strong familial predisposition to eczema, allergic rhinitis, or asthma—and continued supplementation in their infants during the first six months of life—was associated with a marked reduction in the risk of atopic dermatitis (Eslami et al., 2020). The reported decreases in disease incidence were approximately 50, 44, and 36% at two, four, and 7 years of age, respectively (Bager et al., 2003; Illi et al., 2004).

Li A. L. et al. (2017) examined the role of *L. acidophilus* peptidoglycan on immune modulation in the β-lactoglobulin (β-lg) mouse model allergy model with specific reference to the TLR2/NF-κB pathway. Previous work documented an imbalance in the ratio of Treg/Th17 and correlated it with allergic symptomology, wherein Th17-dominated responses play a causative role in allergy. In Li A. L. et al. (2017) study, it was demonstrated that allergy to β-lg enhanced Th17 predominance and that oral administration of the *L. acidophilus* KLDS 1.0738 strain successfully down-regulated such Th17 immunopathologies. This research followed up on that by investigating the function of TLR2 in macrophage recognition of *Lactobacillus* peptidoglycan. Li A. L. et al. (2017) results indicated that peptidoglycan administration triggered macrophage TLR2 expression, which activated downstream NF-κB signaling. Interestingly, inhibition of TLR2 impaired the production of critical cytokines, including IFN-*γ*, transforming growth factor-β (TGF-β), and IL-10, that are typically induced by peptidoglycan. This observation is consistent with earlier reports, e.g., Rakoff-Nahoum et al. (2004) which have documented the protective and restorative functions of TLR signaling in macrophages. Li A. L. et al. (2017) also investigated the function of TLR2 in immunoinhibitory responses induced by *Lactobacillus* peptidoglycan in a β-lg allergy mouse model. Pre-treatment with *Lactobacillus* peptidoglycan resulted in decreased allergy symptoms and elevated levels of TLR2 and NF-κB p65, suggesting a protective function. Notably, MyD88 (a central adapter protein in TLR2 signaling) expression was hardly affected by *Lactobacillus* peptidoglycan, whereas other research has demonstrated that TLR2 ligands also induce MyD88-independent signaling, resulting in NF-κB activation and cytokine induction (Takeuchi et al., 2000; Wang Q. et al., 2001; Kenny et al., 2009). This indicates that *Lactobacillus* peptidoglycan can modulate its immunological activity through MyD88-dependent and independent pathways (Takeuchi et al., 2000).

In addition, intraperitoneal instillation of *Lactobacillus* peptidoglycan in sensitized mice induced concentration-dependent increases in Treg-related cytokines (TGF-β) and transcription factors (Foxp3, CD25), and decreased IL-17A and Retinoid-Related Orphan Receptor gamma t (RORγt) levels (Takeuchi et al., 2000). This indicates that *Lactobacillus* peptidoglycan induces a Treg-biased immune response, which can suppress IgE-mediated allergy. The correlation between TLR2 activation and the balance of Treg/Th17 was also observed, where TLR2 and NF-κB levels were higher with higher Foxp3 and lower RORγt in the high-dose *Lactobacillus* peptidoglycan group than in the allergy group (Takeuchi et al., 2000). In summary, Li A. L. et al. (2017) results indicate that *Lactobacillus* peptidoglycan, via TLR2/NF-κB signaling, enhances the activity of Treg and inhibits Th17-induced inflammation in β-lg-sensitized mice. This study contributes to the literature attesting to the preventive efficacy of probiotics, especially *Lactobacillus*, to reduce allergic inflammation by establishing a Treg-dominant immune microenvironment. In allergic models, signaling pathways downstream of TLRs, including MyD88 and NF-κB, exert context-dependent effects rather than uniformly promoting inflammation. MyD88-dependent signaling can contribute to allergic inflammation when strongly activated; however, under controlled stimulation conditions, it may also support immune regulation by promoting tolerogenic APC functions and regulatory cytokine production.

### 5.4 Cancer

Probiotics are increasingly studied in oncology—not as stand-alone cures, but as adjuncts that influence tumor biology, immunity, and therapy response (Jiang et al., 2024). He et al. (2017) showed the immense anticancer impact of peptidoglycan derived from probiotics and selenylated peptidoglycan (Se-peptidoglycan) on the proliferation and apoptosis of cells in human colon cancer. The studies affirm the production of a dose-dependent antiproliferative action of peptidoglycan and Se-peptidoglycan, with Se-peptidoglycan having an inhibitory activity much greater than that of peptidoglycan. The effectiveness boost by Se-peptidoglycan over peptidoglycan results from the modification of peptidoglycan with selenium, which enhances its anticancer activity (He et al., 2017). Past research has established peptidoglycan’s ability to stimulate an immune response since macrophages stimulated by *Bifidobacterium*-derived peptidoglycan secrete cytokines such as IL-1, IL-6, and TNF-*α* that enhance the killing of tumor cells (Wang L. S. et al., 2001). Choi et al. (2006) also found that *Lactobacillus*-derived peptidoglycan suppressed the growth of colon cancer cells in a time- and dose-dependent manner. Current research furthered the above results by proving that Se-peptidoglycan had three times more inhibitory rate to HT-29 cell growth compared to peptidoglycan, supporting the hypothesis that selenium modification increases the antiproliferative efficacy of peptidoglycan. Besides, the induction of apoptosis is an essential mechanism of cancer cell removal. He et al. (2017) research once again proved that peptidoglycan and Se-peptidoglycan induced apoptosis in HT-29 cells. Apoptotic cell death was also marked by mitochondrial depolarization, intracellular calcium increase, activation of caspase-3, and membrane scrambling. Analysis revealed a significantly greater proportion of cells in the late apoptotic and necrotic phases in the Se-peptidoglycan-treated group than in the peptidoglycan group, suggesting that Se-peptidoglycan exerts a more potent pro-apoptotic effect (He et al., 2017). This implies that selenylation of peptidoglycan increases its capacity to induce apoptosis in cancer cells, and this further confirms the notion that selenium modification enhances the anticancer potency of peptidoglycan (Choi et al., 2006). In summary, these results offer evidence that Se-peptidoglycan, with its increased antiproliferative and pro-apoptotic activities, can be a therapeutic drug for colon cancer treatment. The selenium modification of peptidoglycan is found to remarkably enhance its anticancer activity, and thus it is a valuable research area in cancer immunotherapy and treatment.

Wang et al. (2018) centered on the induction of apoptosis in HT-29 human colon cancer cells by *Lactobacillus paracasei subsp. paracasei* M5 strain peptidoglycan. The result showed that peptidoglycan treatment results in striking changes in the expression levels of apoptosis genes such as *Bcl-xl, Bax, Bad, caspase-3*, and *Cyto-C* (Wang et al., 2018). In a more detailed mechanism, treatment-induced expression of the pro-apoptotic genes *Bax* and *Bad*, as well as repression of the anti-apoptotic *Bcl-xl* gene, is evident. This is indicative of the fact that peptidoglycan tilts pro- and anti-apoptotic signals towards apoptosis. The study further established that peptidoglycan induced typical morphological changes, typical of apoptosis, such as cell shrinking and membrane blebbing, and this was linked to caspase-3 activation. These findings are consistent with earlier results by Yu et al. (2004) as they validated the hypothesis that peptidoglycan induces apoptosis through the activation of caspase-dependent pathways. Apart from that, peptidoglycan treatment caused the release of Cyto-C from the mitochondria into the cytosol, a necessary step in the intrinsic apoptotic pathway (Wang et al., 2018). The release of Cyto-C allows for the activation of caspase-9, which in turn activates caspase-3, a key executioner in apoptosis. This series of events demonstrates the intricate molecular mechanisms in peptidoglycan-induced apoptosis in HT-29 cells (Yu et al., 2004). Overall, the findings indicate that peptidoglycan derived from *L. paracasei* M5 strain causes apoptosis in colon cancer cells via a signal transduction pathway where Bcl-2 family protein regulation and the Cyto-C/caspase-3 pathway are implicated (Yu et al., 2004). These findings point towards the therapeutic role of probiotic-derived peptidoglycan as a cancer treatment.

Nedzvetsky et al. (2020) revealed a dose-dependent action of MPP in glioblastoma cells, which proved that MPP notably increases the expression of PARP1 (Poly(ADP-ribose) polymerase 1) and NF-κB. Muropeptides like MPP have been found to induce innate immune responses and greatly influence various cellular processes such as cytokine secretion, cell survival, and apoptosis (Nedzvetsky et al., 2020). Nedzvetsky et al. (2020) found that MPP treatment in glioblastoma U373MG cells triggers both PARP1- and NF-κB-dependent signaling. They also observed an appreciable loss of migratory capacity and Nicotinamide adenine dinucleotide reduced (NADH) content in the treated cells. NADH reduction in MPP-treated glioblastoma cells is linked to PARP activation, a process that is reported to lower NADH during poly-ADP-ribosylation, a protein-targeting modification that reorganizes DNA (Nedzvetsky et al., 2020). Since sustained activation of PARP can lead to cellular energy homeostasis derangements, activation of PARP-dependent responses can induce cellular dysfunction or death via NF-κB signaling participation (Nedzvetsky et al., 2020). Therefore, the overexpression of PARP and NF-κB reported may have a central function in the augmented reactivity of glial cells in response to MPP treatment. These findings imply that exposure to MPP induces depletion of NADH, hinders migration of cells, and enhances PARP1 and NF-κB expression in glioblastoma cells (Nedzvetsky et al., 2020). Additional studies need to be done to investigate the tumoricidal activity of MPP in glial-derived neoplasms.

## 6 Role of probiotic-derived peptidoglycan in immunotherapy

In recent years, extracellular vesicles (EVs) of probiotic origin have gained increasing attention as key mediators of host–microbe interactions and as promising tools for immunotherapeutic applications (Morishita et al., 2022; Kurata and Uegaki, 2025). Systematic analyses of EVs derived from *Bifidobacterium longum, L. plantarum* WCFS1, and *Clostridium butyricum* have provided critical insights into the immune-activating mechanisms of peptidoglycan (Morishita et al., 2021). Morishita et al. (2021) demonstrated that EV yield and peptidoglycan content do not directly correlate across different probiotic species. Although *C. butyricum* produced the highest number of EVs, the amount of EV-associated peptidoglycan was comparatively low, whereas its cell wall peptidoglycan content was highly enriched. These findings are consistent with earlier observations indicating that EVs possess a molecular composition distinct from that of their parent bacteria, likely due to selective cargo packaging during EV biogenesis (Yang et al., 2018). From a physicochemical standpoint, probiotic-derived EVs are similar in size to mammalian exosomes, making them suitable for both systemic and mucosal delivery (Morishita et al., 2021). Functionally, EVs derived from all three probiotic strains induced robust pro-inflammatory cytokine production—particularly TNF-*α* and IL-6—in macrophages and DCs (Morishita et al., 2021). Given that these probiotics are Gram-positive bacteria, peptidoglycan represents the predominant PAMP responsible for immune activation. Recognition of EV-associated peptidoglycan by immune cells through TLR2 confirms that the immunostimulatory capacity of EVs largely originates from molecular patterns inherited from their parent probiotic bacteria. Importantly, encapsulation of peptidoglycan within EVs confers resistance to degradation by host enzymes, thereby markedly enhancing its immunotherapeutic potential compared with bacterial lysates, which are rapidly degraded by host enzymes and nucleases (Morishita et al., 2021). Further experiments by Morishita et al. (2021) revealed that cellular uptake of probiotic-derived EVs is essential for EV-mediated immunostimulation. EV internalization primarily occurs through clathrin-mediated endocytosis and macropinocytosis, mechanisms similar to those involved in exosome uptake. This process is energy-dependent and does not involve direct membrane fusion, as demonstrated by uptake inhibition at low temperatures. Disruption of these endocytic pathways significantly reduced cytokine production—particularly IL-6—in macrophages and DCs (Morishita et al., 2021). Intracellular processing of EV-associated peptidoglycan was required for immune activation. Notably, inhibition of endocytosis differentially affected TNF-α production in macrophages, suggesting that additional EV cargo components, such as bacterial nucleic acids, may also contribute to cytokine induction via PRRs. Thus, although peptidoglycan serves as the principal immune activator, cooperative signaling among multiple PAMPs within EVs likely shapes the overall immune response (Morishita et al., 2021). From an immunotherapeutic perspective, these findings provide compelling evidence supporting the use of probiotic-derived EVs as immune adjuvants. Effective immunotherapeutic or vaccine responses require early activation of the innate immune system to facilitate antigen presentation and subsequent adaptive immune priming. EV-mediated delivery of peptidoglycan fulfills this requirement by inducing targeted inflammatory responses without the risks associated with live bacterial administration. EVs derived from *C. butyricum* are particularly attractive due to their high production yield, a critical parameter for EV-based therapeutic development. Collectively, these observations position probiotic-derived EVs as efficient and versatile delivery systems for peptidoglycan-based immunotherapy. Their advantages over conventional probiotics and bacterial lysates include favorable physicochemical properties, enhanced stability, and potent immune-activating capacity driven by EV-associated peptidoglycan.

In a subsequent study, Morishita et al. (2022) identified TLR2 as a central mediator of immune responses elicited by probiotic-derived EVs. Peptidoglycan, together with lipoteichoic acids and bacterial lipoproteins, constitutes a major class of TLR2 ligands. Engagement of EV-associated peptidoglycan with TLR2 expressed on immune cells induces robust cytokine secretion, highlighting the pivotal role of TLR2 in sensing probiotic-derived signals. Downstream activation of the JNK/MAPK and NF-κB signaling pathways promotes transcription of pro-inflammatory cytokines such as TNF-α and IL-6, which are essential for early immune responses (Morishita et al., 2022). Notably, TLR2 expression was particularly high on dermal DCs (DDCs), whose activation is known to elicit strong antigen-specific immune responses. These findings underscore the potential of probiotic-derived EVs as natural adjuvants and support their targeted or transdermal delivery as a novel immunotherapeutic strategy.

Probiotic-derived EVs exhibit substantial heterogeneity in size, morphology, and molecular composition, resulting in distinct EV subpopulations with varying immunostimulatory capacities (Zhu R. et al., 2024). Further analysis by Morishita et al. (2022) demonstrated that both the peptidoglycan content of individual EVs and the efficiency of immune cell uptake significantly influence cytokine production. These observations highlight the importance of functionally characterizing EV subgroups to optimize their therapeutic application in EV-based immunotherapies. Although initial concerns were raised regarding the potential effects of TLR2 blockade on EV uptake, experimental evidence showed that EV internalization remained unaffected under TLR2 inhibition, reinforcing the specificity of downstream signaling mechanisms (Morishita et al., 2022). Taken together, these findings strongly support the therapeutic and immunotherapeutic potential of probiotic-derived peptidoglycan, particularly when delivered via an EV platform. Activation of immune cells through TLR2, other intracellular PRRs, and downstream MAPK and NF-κB signaling pathways effectively stimulates innate immune responses while providing sufficient signals to prime adaptive immunity. Compared with whole bacterial cells or bacterial lysates, EV-based platforms offer superior stability, controlled immunostimulation, and enhanced safety. Therefore, probiotic-derived peptidoglycan–loaded EVs represent a highly adaptable and promising strategy for the development of next-generation immunotherapeutic and immune-stimulating agents.

## 7 Conclusion

Accumulating findings demonstrate that distinct probiotics-derived peptidoglycan structures—such as MDP, meso-diaminopimelic acid–containing muropeptides, and anhydro-peptidoglycans—engage PRRs, principally TLR2, NOD1, and NOD2, to elicit divergent immune outcomes. These responses vary according to peptidoglycan chemical composition, bacterial strain origin, cellular target, tissue localization, and disease model. At the level of innate immunity, probiotic-derived peptidoglycan has been shown to modulate macrophage and DC function through TLR2- and NOD-dependent signaling, resulting in either pro-inflammatory cytokine production (e.g., TNF-α, IL-6, NO) or anti-inflammatory regulation characterized by IL-10 induction and suppression of IL-12/IL-23 pathways. Importantly, protective effects in experimental colitis models are primarily supported by NOD2-dependent mechanisms, including localized IL-10 production, induction of tolerogenic CD103^+^ DCs, and expansion of Foxp3^+^ Tregs, rather than by generalized immune suppression. In adaptive immunity, peptidoglycan recognition via NOD1 and NOD2 influences Th cell polarization in a ligand- and co-stimulation–dependent manner. In the absence of concurrent TLR signals, peptidoglycan-NOD interactions tend to favor Th2-skewed or regulatory responses, whereas combined peptidoglycan and TLR engagement—such as during infection—can amplify Th1 and Th17 immunity. These findings underscore that peptidoglycan does not intrinsically promote a fixed Th1/Th2/Th17 balance, but instead shapes adaptive immune outcomes through receptor synergy and microenvironmental cues.

Evidence supporting therapeutic relevance is strongest in well-defined preclinical settings, including murine models of IBD and infection. In contrast, claims regarding peptidoglycan efficacy in cancer or systemic inflammatory disorders should be interpreted cautiously, as they rely on limited experimental models and specific peptidoglycan preparations rather than broad clinical validation. In conclusion, probiotic-derived peptidoglycan should be regarded not as a uniform immunomodulator, but as a structurally diverse class of postbiotic molecules with receptor-specific and disease-restricted effects. Future translational progress will depend on precise peptidoglycan structural characterization, standardized purification methods, and rigorous validation in disease-relevant models. Such an approach is essential to rationally exploit peptidoglycan-based immunomodulation while avoiding unsupported generalizations across unrelated pathological conditions.
